# Towards Automated Annotation of Benthic Survey Images: Variability of Human Experts and Operational Modes of Automation

**DOI:** 10.1371/journal.pone.0130312

**Published:** 2015-07-08

**Authors:** Oscar Beijbom, Peter J. Edmunds, Chris Roelfsema, Jennifer Smith, David I. Kline, Benjamin P. Neal, Matthew J. Dunlap, Vincent Moriarty, Tung-Yung Fan, Chih-Jui Tan, Stephen Chan, Tali Treibitz, Anthony Gamst, B. Greg Mitchell, David Kriegman

**Affiliations:** 1 Department of Computer Science and Engineering, University of California, San Diego, La Jolla, CA, United States of America; 2 Department of Biology, California State University, Northridge, Northridge, CA, United States of America; 3 Biophysical Remote Sensing Group, School of Geography, Planning and Environmental Management, University of Queensland, St. Lucia, QLD, Australia; 4 Integrative Oceanography Division, Scripps Institution of Oceanography, University of California, San Diego, La Jolla, CA, United States of America; 5 Catlin Seaview Survey, Global Change Institute, University of Queensland, St. Lucia, QLD, Australia; 6 Joint Institute for Marine and Atmospheric Research, University of Hawaii at Manoa, Honolulu, HI, United States of America; 7 National Museum of Marine Biology and Aquarium, Checheng, Taiwan, Republic of China; 8 Charney School of Marine Sciences, University of Haifa, Haifa, Israel; 9 Department of Neuroscience, University of California, San Diego, La Jolla, CA, United States of America; Biodiversity Research Center, Academia Sinica, TAIWAN

## Abstract

Global climate change and other anthropogenic stressors have heightened the need to rapidly characterize ecological changes in marine benthic communities across large scales. Digital photography enables rapid collection of survey images to meet this need, but the subsequent image annotation is typically a time consuming, manual task. We investigated the feasibility of using automated point-annotation to expedite cover estimation of the 17 dominant benthic categories from survey-images captured at four Pacific coral reefs. Inter- and intra- annotator variability among six human experts was quantified and compared to semi- and fully- automated annotation methods, which are made available at coralnet.ucsd.edu. Our results indicate high expert agreement for identification of coral genera, but lower agreement for algal functional groups, in particular between turf algae and crustose coralline algae. This indicates the need for unequivocal definitions of algal groups, careful training of multiple annotators, and enhanced imaging technology. Semi-automated annotation, where 50% of the annotation decisions were performed automatically, yielded cover estimate errors comparable to those of the human experts. Furthermore, fully-automated annotation yielded rapid, unbiased cover estimates but with increased variance. These results show that automated annotation can increase spatial coverage and decrease time and financial outlay for image-based reef surveys.

## Introduction

Coral reefs provide habitat to a wide diversity of organisms, and substantial economic and cultural benefits to coastal communities [1,2]. These functions are threatened by global declines in coral cover caused by a wide diversity of natural and anthropogenic disturbances including global climate change and ocean acidification [3]. The decline in coral cover has been dramatic, with > 80% decrease in the Caribbean over the last four decades [4], and 1–2% loss each year in the Indo-Pacific between 1997 and 2003 [5]. These rates of decline are forecast to increase [6], and extensive surveys are urgently needed to better understand the “coral reef crisis” [7] and contextualize effective ecosystem-based management for this critical ecosystem [8].

Reef surveys have traditionally been performed *in situ* by scuba divers skilled in marine ecology and capable of identifying and counting taxa underwater. *In situ* surveys enable accurate observations, but they are time-consuming and allow only small areas of the reef to be surveyed. Additionally, they are dependent on the skill of the experts conducting the surveys, and the data are usually not available for re-analysis. *In situ* surveys were largely replaced by image-based surveys when high quality underwater cameras became available at an economic price in the early 1960’s [9], coincident with the proliferation of scuba as a tool for underwater research. Image-based surveys are advantageous as they allow faster data collection and provide a permanent record that can be analyzed for organism abundance [10] and demographic properties [11]. However, quantifying organisms in benthic photographs is more challenging than *in situ* inspection, due to limited image resolution, variable lighting conditions, water turbidity, and the inability to interact physically with the benthos. Although some efforts have been made to quantify accuracy and inter annotator variability in surveys conducted using underwater video transects [12,13], there is little information describing the variability associated with annotations of coral reef survey images.

Since the turn of the new millennium, the application of image-based tools for marine ecology has changed dramatically in three domains. First, improvements in digital photography have allowed large numbers of images to be gathered at increasing resolution. Second, the capabilities of computers and software to manipulate, store, and analyze images have increased by orders of magnitude. Third, advances in robotics and control theory have enabled the construction of autonomous underwater vehicles (AUVs), remotely operated vehicles (ROVs), and imaging sleds that can capture thousands of survey images in a single deployment [14–17]. However, the capacity to analyze images has not advanced at a pace commensurate with the capacity to collect them. This has created a ‘manual-annotation’ bottleneck between the rapid image acquisition and the quantitative data needed for ecological analysis.

Generating quantitative ecological information from underwater images typically involves random point annotation to estimate the percent cover of the substrata of interest. To generate these data, substratum types are identified, usually manually by an expert in that ecosystem, for a number of randomly selected locations in each image. Statistically valid sampling of benthic habitats with image- and point- based tools requires careful attention to the primary purpose of the study, choice of the experimental and statistical approaches necessary to answer the questions being addressed, and the statistical power (i.e., a function of sample size, variance, and desired difference to be detected) required to test the hypotheses of interest. These experimental design components are not the focus of this paper, and interested readers are referred to the many excellent texts on these subjects [10,18]. Instead, we focus on the means and accuracy by which quantitative information can be manually and automatically extracted from underwater images using random point annotation, specifically for near-shore tropical marine environments.

Point annotations are typically preformed using manual annotation software like Coral Point Count with Excel Extensions (CPCe) [19], photoQuad [20], pointCount99 [21], Biigle (biigle.de) or Catami (catami.org), which facilitate the annotation process by providing user interfaces and tools for the export of relevant data. Recently, there have been several efforts to automate point annotation of benthic survey images [22–26], but the implementation of these tools has been hindered by two issues. First, while human experts have been annotating benthic images for decades, the accuracy of this procedure (i.e., intra-annotator error) remains largely unknown, as does the extent to which results varies between annotators (i.e., inter-annotator error). This information is critical as it provides a baseline against which the efficacy of current and future automated annotation methods can be assessed. Second, there is scope to develop “hybrid” annotation modes, which automatically annotate a significant portion of the data, but defer the most uncertain identification decisions to human annotators. The value of such modes will depend on the quantitative means by which “uncertain” is evaluated, and on the appropriate trade-off between accuracy and efficiency for the question at hand.

In this paper we focus on codifying the criteria for implementing an existing automated image analysis tool [24] for coral reef survey images. Specifically, we sought to answer two questions: (1) what is the baseline variation among human experts in the analysis of benthic communities that we would hope to equal or improve upon through automated methods, and (2) what is the appropriate framework for optimizing the trade-off between the high accuracy of a human annotator and the high efficiency of an automated annotator? To answer the first question, we designed a study in which we quantified the variability among multiple experts in the analysis of benthic images from coral reefs in Moorea (French Polynesia), Nanwan Bay (Taiwan), the northern Line Islands, and Heron Reef (Great Barrier Reef), and compared their results to those obtained through computer-based automated analysis. To address the second question, we then altered the requirements for more (or less) human supervision in the modes of annotation in order to quantify the trade-off between accuracy and efficiency. Finally, a key contribution of this work is that the methods developed for automated annotations are incorporated in the random point annotation tool of CoralNet (coralnet.ucsd.edu), which is publically available.

## Materials & Methods

Digital photoquadrats from coral reefs in four locations throughout the Pacific, with 671–3472 images per location, were used to provide a diverse model system for testing our analytical tools (Table 1). These images were originally annotated by experts using 24–200 random point locations superimposed on each image. From each image set at each location, 200 images were randomly selected and designated as the Evaluation set; the remainder were designated the Reference set. The four Evaluation sets were then re-annotated independently, and without prior knowledge of the initial annotations, by each of six human experts at 10 point locations in each image, sub-sampled at random from the original point locations. Following the human annotation, an implementation of a computer vision algorithm [24] was then used to automatically annotate the same 10 points per image. The four Reference sets were used to train the automated annotator, and were made available as training material for the human experts. The multiple sets of annotations were used to evaluate intra- and inter-annotator variability and to evaluate the proposed operational modes of automated annotation. All images and data used in this study are made available at (doi:10.5061/dryad.m5pr3).

### Study locations

Digital photoquadrats were obtained from projects monitoring coral reef community structure in the outer and fringing reefs of Moorea (French Polynesia), Kingman, Palmyra, Tabuaeran and Kiritimati atolls (northern Line Islands), Nanwan Bay (Taiwan), and the platform reefs at Heron Reef (Great Barrier Reef, Australia). These study locations were selected because they offered legacy data involving large numbers of images that had been annotated with equivalent random point methodologies by experts with extensive experience in identifying benthic organisms from photographs at their respective locations. In each location, multiple species of scleractinian corals, macroalgae, crustose coralline algae, and various non-coral invertebrates densely populate benthic surfaces, and photoquadrats are characterized by complex shapes, diverse surface textures, and intricate boundaries between dissimilar taxa. Additionally, water turbidity and light attenuation degrade colors and image clarity to varying degrees for the four image sets, presenting a challenging task for both manual and automated annotation. It should be noted however, that these are all typical conditions, and these image sets represent typical survey images taken for purposes of coral reef ecology.

**Fig 1:**
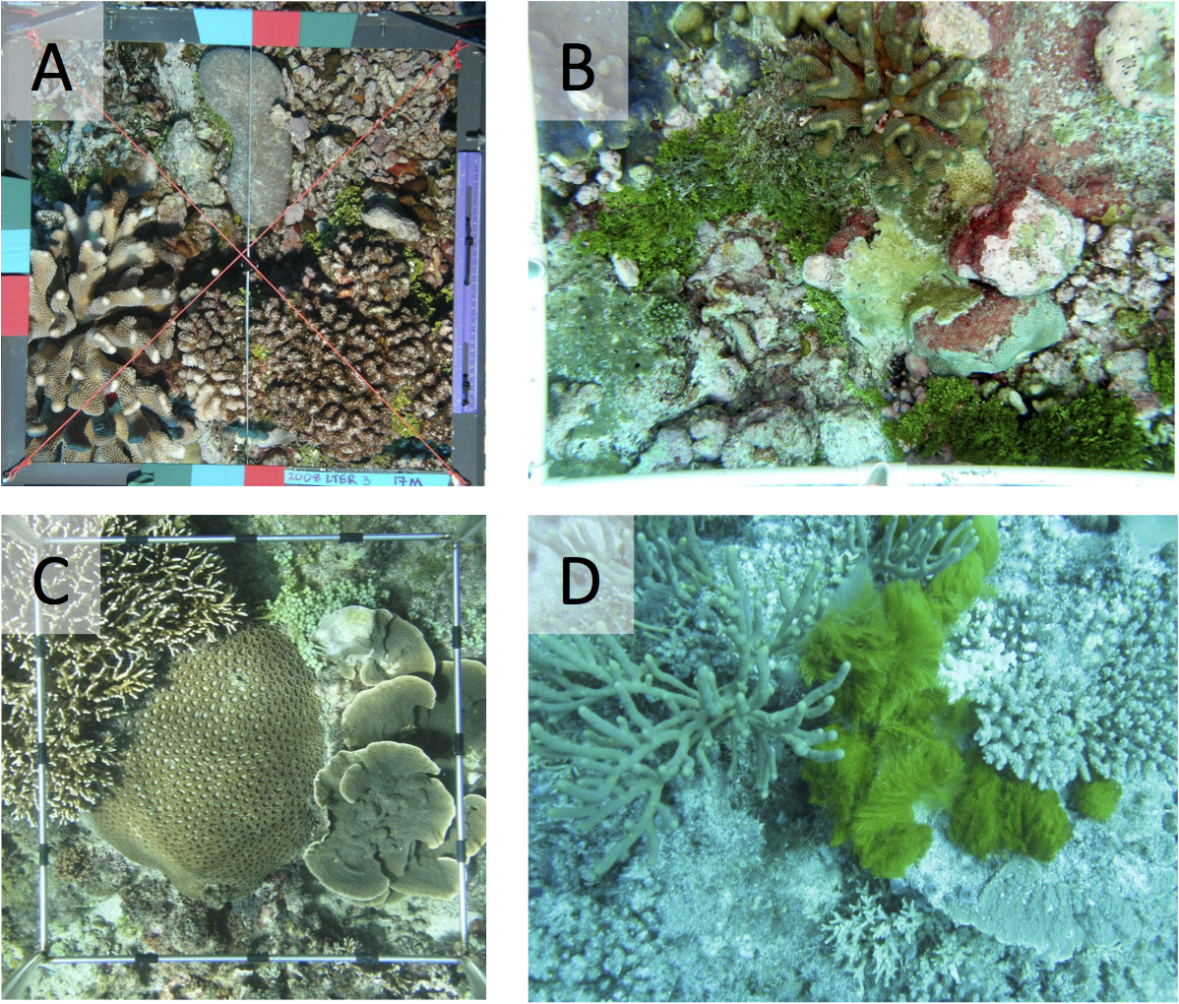
Sample photoquadrats. Sample photoquadrats acquired and annotated as part of long-term monitoring projects on shallow coral reefs (≤ 17-m depth). (A) Moorea, French Polynesia, acquired with strobes and framer (50 x 50 cm); (B) Palmyra, northern Line Islands, acquired using a manual white balance and framer (65 x 90 cm); (C) Nanwan Bay, Taiwan acquired with framer (35 x 35 cm) but neither strobes nor white balance; (D) Heron Reef, GBR, acquired without framer, strobes or white balance.

The four locations also represent the great variation commonly found within and among photographic surveys of shallow (< 20 m depth), Pacific coral reefs. This variation includes differences among locations in species diversity and their colony morphologies, variation in camera equipment (e.g., angle of view, and resolution), distance between camera and benthos (and whether the distance was constant among photographs), and the mechanism employed to compensate for the depth-dependent attenuation of sunlight (i.e., through the use of strobes and/or manual white balance adjustment of camera exposures). The photographs from Moorea, the Line Islands and Nanwan Bay were recorded using framers to hold the camera perpendicular to, and at a constant distance from, the sea floor. Underwater strobes were used in Moorea to restore surface color and remove shadows from images, and in the Line Islands, image-colors were adjusted through manual adjustment of the white balance for each series of images. Neither strobes nor color correction were used to record photoquadrats in Nanwan Bay. Finally, at Heron Island, the reef was recorded using a camera (without strobes or white balance correction) that was hand-held above the reef using a weighted line suspended below the camera to maintain an approximately fixed distance to the sea floor [27]. Refer to Fig 1, and S1 Fig for sample images from the locations, to Table 1 for a data summary, and to S1 Appendix for additional details on the survey locations.

**Table 1:** Summary of image resources.

	Moorea	Line Islands	Nanwan Bay	Heron Reef
Investigator	P.J. Edmunds	J. Smith	T-Y Fan	C. Roelfsema
Sampling year	2008	2005	2012	2007
Reef type	Fringing reef	Fringing reef	Fringing reef	Platform reef
Geomorphic zone (Photo depth)	Fringing reef (2–5 m), Fore reef (10 m & 17 m)	Reef flat / lagoon (2–5 m)	Fore reef (2–5 m)	Fore reef (5 m) Reef flat (1 m)
Image cover (cm)	50 × 50	65 × 90	35 × 35	50 x 65
Image size (px.)	6.24 M	7.1 M	9.98 M	6.2 M
Spatial res. px. (mm^2^)	24.96	12	81.46	19.08
Camera	Nikon D70	Olympus 7070	Canon G12	Canon A540
Lighting	Dual Nikonos SB 105 strobes	No lighting, manual w.b.	No lighting	No lighting
Evaluation set				
# images	200	200	200	200
# anns. image^-1^	10	10	10	10
# total anns.	2,000	2,000	2,000	2,000
Reference set				
# Images	471	532	690	2,597
# anns. image^-1^	200	100	50*	24
# total anns.	94,200	53,200	34,260	62,328

Tabulated information about the four original reef-surveys used in this study.

# = number of, anns. = annotations, M = million, res. = resolution, px. = pixel, w.b. = white balance,

* Some images are only annotated with 49 points.

### Label-set

When this study began, the photoquadrats from each of the four locations had already been manually annotated by the local coral reef experts using four different label-sets defined by the respective experts. To make comparisons of annotator accuracies between locations, a consensus label-set was created to which the four original label-sets were mapped (Table 2). The consensus label-set consisted of 8 scleractinian genera and 1 ‘other scleractinians’ label; 3 algal functional groups (macroalgae, crustose coralline algae (CCA), and turf algae); and 9 other labels that included sponges, sand, and the hydrozoan *Millepora*. In our analysis we also considered all coral labels together as a coral functional group. The labels of the consensus label-set were chosen to enable a one-to-one or many-to-one mapping from the original label-sets in Moorea, Line Islands, and Nanwan Bay. Corals were not resolved to genus level in the original Heron Reef label-set and all Archived coral annotations were therefore mapped to the generic ‘other scleractinians’ label for this location.

**Table 2:** Consensus label-set.

Label	Definition
*Acropora*	Coral genus
*Favia*	Coral genus
*Favites*	Coral genus
*Montastraea*	Coral genus
*Pavona*	Coral genus
*Platygyra*	Coral genus
*Pocillopora*	Coral genus
*Porites*	Coral genus
Other scleractinians	Other hard corals
*Millepora*	Genus of hydrozoan coral
Sponges	All types of sponges
Soft Coral	All soft corals
Crustose coralline algae	All genera
Turf algae	Here defined as multi-specific assemblages: 1 cm or less in height
Macroalgae	All genera—defined as larger algae > 1 cm in height
Sand	Sand, silt or other fine-grained, soft substratum
Bare space	Rock, Basalt, Limestone, Dead coral, Rubble, or other hard substratum. Note, use this label only if *not* overgrown by algae
Transect hardware	Transect line, wand, and framer. Anything that is part of the sampling methodology
Unclear	Dots falling in shadowy, dark or blurry areas, where a class designation is not possible
All other labels	Any substratum not covered by the other labels, e.g., seagrass, other invertebrates, terrestrial trash

Label-set used in this work, and definition provided to human annotators.

### Manual Annotations

As part of the present analysis, the photoquadrats from each of the four locations were manually re-annotated by six human experts. The local expert familiar with each location were designated as the ‘Host’, and the other five experts with less familiarity with the specific locations were designated ‘Visitors’. All six were experts in identify corals and benthic taxa at coral reefs in the tropical Pacific Ocean. The annotations completed by the Hosts prior to the present study as part of the original ecological analyses were denoted ‘Archived’. One to six years had passed since the original annotations were made, and thus we reasoned that the Hosts would not be biased by their own original annotations. We emphasize that there are four Hosts in this study; one for each location, and that the Hosts for each location re-annotated the same points in the same images that they had annotated previously themselves. Intra-annotator variability could thus be measured by comparing the Host and Archived annotations. All of the present annotations were performed using the random point annotation tool of CoralNet (S2 Fig). To assist the experts in the new annotations, the images and Archived annotations of the Reference sets from each location were stored in CoralNet and used as a virtual learning tool to improve identification of the benthic taxa (S2 Fig). All images were scored using 10 points per image, so with 200 images per location and 4 locations, each annotated by 6 experts, this study generated 48,000 manual point-annotations. The manual annotation effort required on average 1 minute per image, for a total annotation time of approximately 15 hours per expert. This annotation time was divided into several 1–3 hour sessions over 1–4 weeks depending on the preference of the annotator.

### Automated Annotations

We previously developed an automated annotation system for coral reef survey images [24]. In this system, the texture and color of a local image patch around a location of interest (i.e., one of the randomly selected points) is encoded as a count of ‘visual words’, which is a quantization of the visual appearance space of the image. The encoded visual information is then used, together with a set of labels, to train the automated annotator, using the Support Vector Machines (SVM) algorithm [28].

The method of [24] was modified in two ways to increase the accuracy and reduce the runtime. First, the vector quantization step of [24] was replaced by Fisher Encoding [29] which was recently shown to improve classification accuracy for various image classification tasks [30]. Second, the kernel-based SVM of [24] was replaced by a linear SVMs which have significantly lower runtime [28–31]. The final method used in this work required 20 seconds to pre-process each image, the training of the SVM required 5–20 minutes for each location depending on the size of the Reference set for that location, and automated annotations of an unknown image from the Evaluation set required < 1 second. The aforementioned times were measured on a single computational core.

The color and texture information of the images in the Evaluation and Reference sets was encoded as a 1920 dimensional ‘feature’ vector for the annotated point in each image [29]. The feature vectors from the Reference set were paired with the Archived annotations to form a training-set, which was used to train a one-versus-rest linear SVM (S1 Appendix). This training was performed separately for each location so that the training-set from each location was used to train a SVM, which was then used to automatically annotate the images in the Evaluation set from the same location.

Since the images in the Evaluation set were selected randomly from the set of available images, the mean percent cover of each label was similar in the Reference and Evaluation sets. This means that approximately correct cover estimates of the Evaluation set could be generated by randomly annotating point locations in the same proportions as is present in the Reference set. However, in other situations where the automated annotator is trained on data from, for example, a previous year or another location, the proportions of the different types of annotations (e.g., percent coral) will likely differ between training data (here: Reference set) and test data (here: Evaluation set). The following pre-processing step was applied to ensure that any conclusions from this study, with respect to the efficacy of the automated annotator to estimate percent covers, would be valid in such situations. In this pre-processing step, a randomly selected subset of the non-coral annotations in the Reference sets was discarded, so that the proportion of coral annotations increased by 10%. For example, there were 19,566 coral, and 74,634 non-coral annotations in the Moorea Reference set, and therefore, 20.77% was coral. A random subset of 8,566 non-coral annotations was discarded so that 66,068 non-coral annotations remained. This resulted in a 10% increase in the estimate of coral abundance (22.85%).

### Post-processing of annotations

Two inconsistencies in the Hosts’ and Visitors’ annotations were noted and corrected. First, some expert annotators mapped points on the images that fell on transect hardware to the ‘Transect hardware’ label, while others mapped them to substratum types that were inferred to occur beneath the hardware based on close proximity adjacent to the hardware. Such discrepancies were due to incomplete instructions to the experts rather than identification difficulties. Therefore, any point locations that two or more of the experts mapped to ‘Transect Hardware’ were considered to be transect hardware, and all annotations for that point location were changed to ‘Transect hardware’ in post-processing.

The second inconsistency arose with the scoring of images from Moorea because neither the original label-set in this location, nor the Archived annotations, contained the ‘Bare space’ label of the consensus label-set. Instead, in this location all “bare space” was effectively covered with CCA and therefore was mapped to a ‘CCA’ label. However, in the annotation of the present study, some experts mapped these areas to ‘Bare space’ as the best match to this state in the consensus label-set. To facilitate a comparison between the multiple sets of annotations, all ‘Bare space’ annotations for the Moorea location were changed to ‘CCA’ in post-processing.

### Estimating Annotator Variability

Using the Archived annotations as baseline, the accuracy of annotator *a* was estimated using the Cohen’s kappa statistic, and denoted *κ* ^*a*^ [32]. Additionally, $\kappa_{\Psi }^{a}$ denoted the Cohen’s kappa of the binary classification task between a set of labels, Ψ (e.g., corals, macroalgae, or *Acropora*), and the labels not in Ψ. For example, $\kappa_{\text{coral}}^{\text{Host}}$ denoted the Hosts’ accuracy for the task of discriminating between coral and non-coral. For notational brevity, the super-script is henceforth dropped when it is clear from the context which annotator is considered. A one-sample Kolmogorov-Smirnov test was performed to determine normality of *κ*. This test yielded p-values < 0.001 for all labels, annotators and locations, indicating non-normality and the consequent need for non-parametric tests. Differences in *κ* (using the full label-set) between the Hosts and Visitors were assessed using non-parametric Mann-Whitney U tests, and difference between the four functional groups (coral, macroalgae, CCA, and turf algae) was assessed using a Kruskal-Wallis test, both using the four locations as repeated trials.

Additionally, using again the Archived annotations as baseline, a confusion matrix, *Q* was estimated for each location and annotator. Each confusion matrix *Q* was 20 rows by 20 columns and values at row *r*, column *c* in the matrices indicate the ratio of annotations originally labeled by Archived as label *r* now classified by the Host, Visitors, and automated annotator, respectively, as label *c*.

### Modes of Automated Annotation

In this section, the proposed operational modes for semi- and fully- automated annotation are detailed.

#### The Alleviate Operational Mode

A one-versus-rest automated classification procedure generates a vector of classification scores corresponding to each classification decision [33]. The score vector can be used to estimate the certainty of the automated classifier for an unseen example [34]. For example, if all scores are low and similar to each other, the classifier is more likely to assign the incorrect label [34]. In such situation, an alternative is to let the classifier abstain from making an automated annotation decision and instead defer to a human expert. Such procedure enables a trade-off between the amount of human effort and the accuracy of the final annotations.

The classification scores were used in an operational mode, which we denote ‘Alleviate’ because it alleviates the workload for the human annotator. The scores were denoted *s*_*i*,*k*_(*m*) where subscript *i* indicate the image, subscript *k* the point location, and *m* the class label. For a given point in an image in the Evaluation set the vector of 20 scores, one for each class, was denoted [*s*_*i*,*k*_]. Using Alleviate, an automated annotation was only assigned when, for a given point location, there existed a score *s*_*i*,*k*_(*m*) such that *s*_*i*,*k*_(*m*) > *ϵ*, for some threshold, *ϵ*. More formally, the Alleviate annotations, $y_{i,k}^{\text{Alleviate}}$ were defined as:

$$y_{i,k}^{\text{Alleviate}}=\{\begin{array}{l} \;y_{i,k}^{\text{Automated}}\;\text{if max}[s_{i,k}]>\epsilon \\ \;y_{i,k}^{\text{Host}}\;\text{otherwise} \end{array}$$

where $y_{i,k}^{\text{Automated}}$ was the automated annotation of point *k* in image *i*, and $y_{i,k}^{\text{Host}}$ the corresponding Host annotation. We denoted by *λ*(*ϵ*) the ‘level of alleviation’, the fraction of samples classified by the automated annotator for a certain threshold, *ϵ*. A level of alleviation of *λ*(*ϵ*) = 50% was used in the final analysis of Alleviate.

The same alleviation procedure was also used, in one analysis, to combine the Visitors’ and the automated annotations. However, unless explicitly stated otherwise, Alleviate denoted the combination of the Hosts’ and the automated annotations throughout this paper.

#### The Abundance Operational Mode

While the automated annotations are, in general, less accurate than those provided by human experts [23,24,26], they have the benefit of being, in contrast to a human expert, deterministic, meaning that the automated annotator will always make the same classification decision for the same image. If the confusion matrix is known for the automated annotator, it can be used to generate annotations that are accurate in aggregate (such as abundance of taxa) even though individual decisions might be erroneous [35,36]. The corrected abundances will, by design, be unbiased, but with larger variation than those based on the automated annotations [35].

Using the abundance correction method of [35,36], a fully automated deployment mode, Abundance was defined. In this mode, cover estimates for each image *i* were calculated as:

$$c_{i}^{\text{Abundance}}(m)={\left(Q'\right)}^{-T}c_{i}^{\text{Automated}}(m)$$ (1)

where $c_{i}^{\text{Automated}}(m)$ denoted the cover in image *i* of class *m* based on the automated annotations, *Q*' was a confusion matrix estimated through a 20 fold cross-validation on the Reference set, and the–T superscript denotes matrix transpose followed by inverse. Note the difference between *Q*', which was estimated for the automated annotator from the Reference set and used in Abundance, and *Q*, which was estimated for all annotators from the Evaluations sets and used to evaluate the final performance.

### Performance evaluation based on cover estimates

The estimates of ecological composition based on the operational modes were contrasted with the estimates based on the Archived, Hosts’, and Visitors’ annotations. The cover of label *m*, for image *i*, was denoted $c_{i}^{\text{a}}(m)$ for each annotator (or mode):

a∈{Archived,Host,Visiting1,…,Visiting5,Alleviate}

In addition, $c_{i}^{\text{Abundance}}$ was derived from the automated annotations using Eq 1. The pairwise differences from the Archived cover estimates for each image were denoted:

$$d_{i}^{a}(m)=c_{i}^{a}(m)-c_{i}^{\text{Archived}}(m)$$

The average estimation error (bias) for annotator (or mode) *a*, label *m* for the 200 images in a certain location was denoted:

$$e^{a}(m)=\frac{1}{200}\overset{200}{\underset{i=1}{\sum }}d_{i}^{a}(m)$$

The Mean Absolute Error (MAE) was denoted as the mean absolute value of *e* ^*o*^(*m*) calculated for a particular substratum and group of annotators across the four locations (e.g., the macroalgae cover as estimated by the Hosts).

The differences $d_{i}^{a}(m)$ were also used to test the null hypothesis that, for each label, annotator and dataset, the mean of $d_{i}^{a}(m)$ is zero. A one-sample Kolmogorov-Smirnov test was first performed to determine normality of $d_{i}^{a}(m)$. This test yielded p-values < 0.001 for all label (or label groups), annotations, and locations, indicating non-normality and the consequent need for a non-parametric test. A permutation t-test was therefore performed at the 95% significance level with a Bonferroni correction for eight comparisons (Alleviate, Abundance, Host, and 5 Visitors) [37]. The differences $d_{i}^{a}(m)$ were also used to estimate a 95% confidence interval for all contrasts using the percentile-t bootstrap procedure [37].

### CoralNet

As a key contribution of this work, we make implementations of the described modes of operation (Alleviate, Abundance, and Refine ( S1 Appendix )) available on CoralNet (coralnet.ucsd.edu). CoralNet is a software module that has been designed as a repository and online annotation tool for benthic survey images, and it allows users to upload and annotate survey images with a user-defined label-set. Annotations are performed using a random point annotation interface similar to that of CPCe [19]. CoralNet also offers tools for browsing images and annotations as well as for viewing and exporting estimated cover statistics. The implementation of Alleviate allows users to determine the level of alleviation based on the accuracy of the automated annotator, and Refine is implemented with 5 suggested labels per point location.

## Results

### Annotator Accuracy

#### Substratum identification accuracies

**Fig 2:**
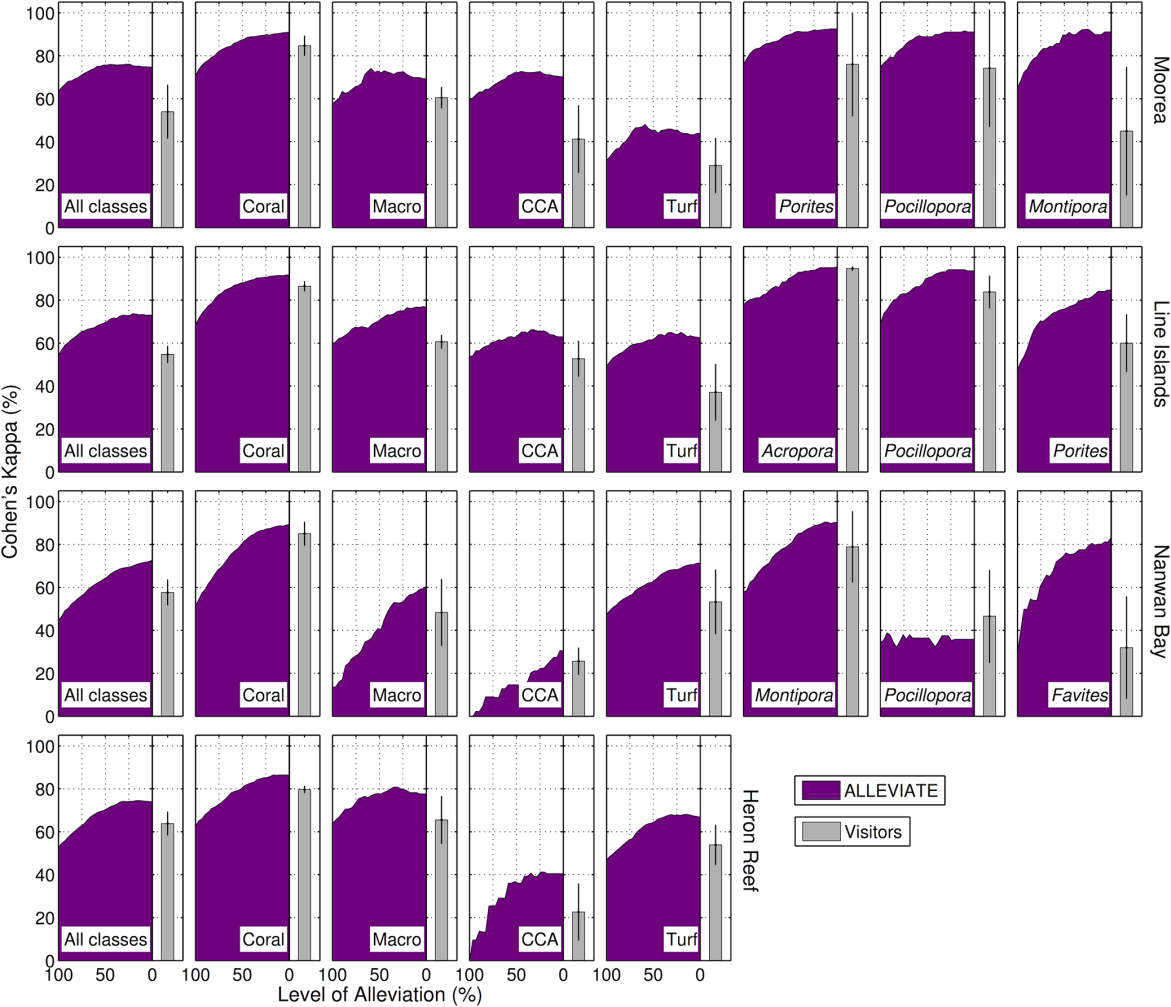
Annotation accuracy. Accuracy as measured by Cohen’s kappa for the Hosts’ annotations with various level of alleviation, and for the Visitors’ (mean ± SD, n = 5) annotations. The left edge of the alleviation curve corresponds to automated annotations only, and the right edge corresponds to the Hosts’ annotations only. The first column shows accuracy calculated from the full label-set. Column two to five show accuracy for the task of discriminating functional groups: coral, macroalgae, crustose coralline algae (CCA), and turf algae, respectively, versus the rest. Columns six, seven and eight show accuracy for the three dominant coral genera. Genus-level identification for Heron Reef is not provided, as Archived annotations were not available to this resolution.

Annotator accuracy was measured as Cohen’s kappa, *κ* of the annotators, compared to the Archived annotations. The Hosts’ accuracies differed among the functional groups (H = 10.08, df = 3, p = 0.018). Specifically, the accuracy for each functional group was: *κ*_coral_ = 89.7±1.2%, *κ*_macroalgae_ = 71.0±4.0%, *κ*_CCA_ = 51.0±9.3%, and *κ*_turf algae_ = 61.6±6.1% (mean ± SE, n = 4 locations, Fig 2, S1 Table), with the majority of confusion occurring among algal groups (S3 Fig). The Hosts’ accuracy for common coral genera (i.e., with > 10 Archived annotations) was *κ*_coral genera_ = 79.4±4.2% (n = 19, S1 Table). Coral genera were most commonly confused with other coral genera, in particular with the general label of ‘other scleractinian’. However, there was also notable confusion between the turf algae and CCA (S3 Fig).

#### Relationship between abundances and identification accuracies

**Fig 3:**
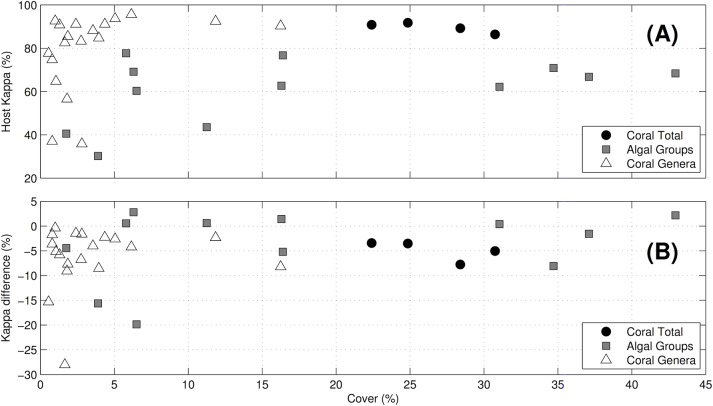
Comparison between accuracy and percent cover. Comparison between mean percent cover and annotation accuracies displayed as (A) Cohen’s kappa for the Hosts, and (B) Difference in Cohen’s kappa between Alleviate and the Hosts, plotted against the percent cover of coral genera and functional groups. Data is drawn from all four locations except for the coral genera where data was not available for Heron Reef.

The Hosts’ accuracies were not correlated with the benthic covers (r = 0.089, df = 33, p = 0.61), although there were a few outliers recorded a low (< 5%) cover (Fig 3A). The annotation accuracy of corals (when considered as a group) was > 80% in all four locations; accuracy of algal groups was > 60% except for three samples with < 12% cover; and accuracy of coral genera was generally > 80%, except six samples with < 3% cover.

#### Inter-annotator variability

The Visitors’ annotation accuracies were lower than the Hosts’ (U = 26, n = 4, p = 0.029). Specifically, the accuracy for each functional group was: *κ*_coral_ = 84.0±1.0%, *κ*_macroalgae_ = 58.7±2.5%, *κ*_CCA_ = 35.5±3.7%, and *κ*_turf algae_ = 43.3±3.6% (mean ± SE, n = 20, Fig 2, S1 Table). The Visitors’ accuracy for common coral genera (i.e., with > 10 Archived annotations) was *κ*_coral genera_ = 58.6±2.9% (n = 95, S1 Table). As with the Hosts, the principal confusion occurred among the algal groups, and the principal confusion among coral genera occurred with the ‘other scleractinian’ label (S3 Fig). The lower accuracy of the Visitors was evident also in the lesser weight across the confusion matrix diagonals compared to the matrix diagonals of the Hosts (S3 Fig).

### Modes of Automated Annotation

The accuracy of the automated annotation method was: *κ*_coral_ = 63.5±4.3%, *κ*_macroalgae_ = 48.5±11.7%, *κ*_CCA_ = 28.3±16.3%, and *κ*_turf algae_ = 43.8±4.2% (mean ± SE, n = 4, Fig 2, S1 Table). This drop in accuracy compared to the Hosts (e.g., over 20% for *κ*_coral_) suggested that a direct application of the automated annotation method of [24] would not yield reliable cover estimates of benthic substrata. Instead, performance of the automated annotation method was evaluated within the context of the proposed operational modes.

#### Semi- automated annotation using Alleviate

**Fig 4:**
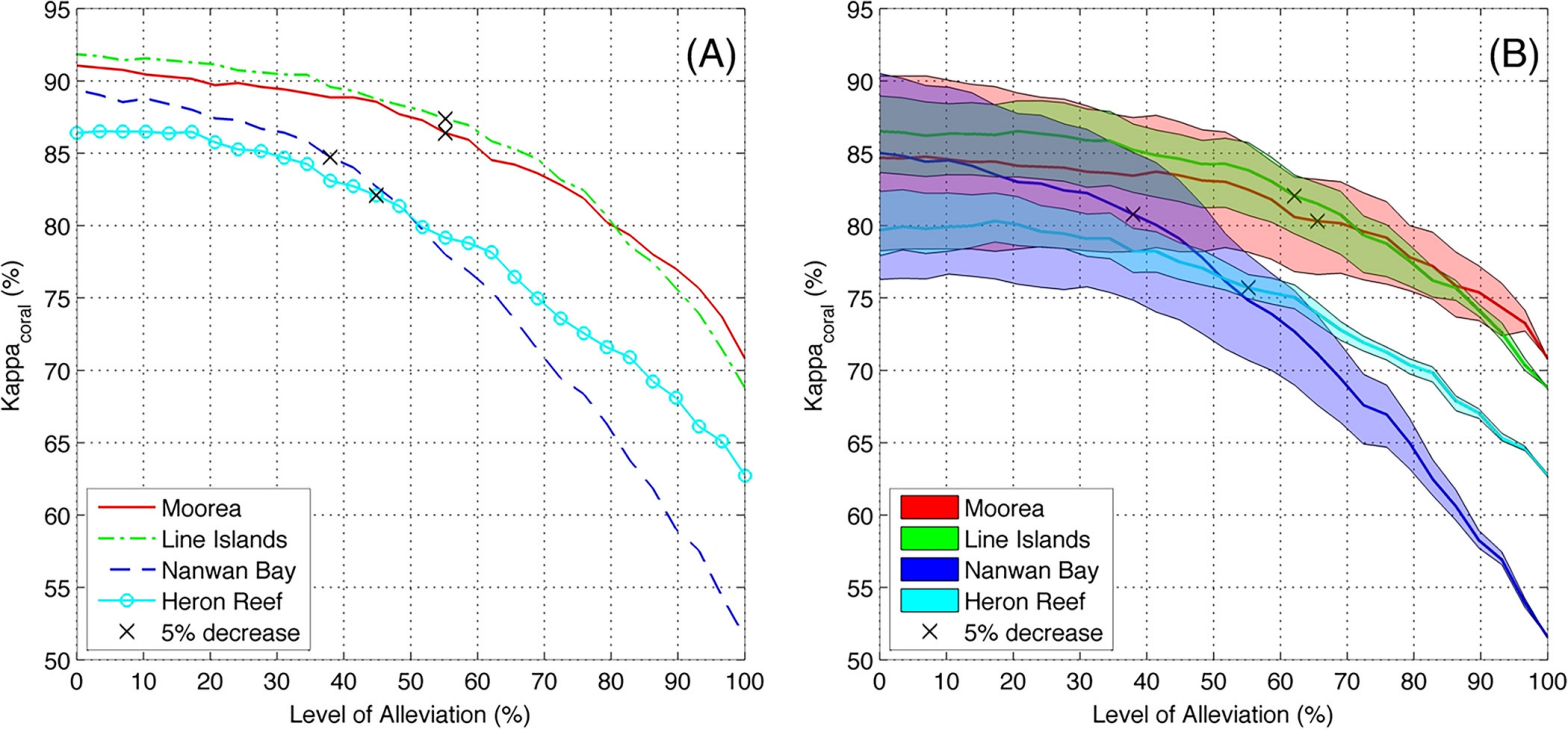
Alleviation levels for Hosts and Visitors. Accuracy as measured by Cohen’s kappa for the task of discriminating corals from other labels, *κ*_coral_ at various levels of Alleviation for the four studied locations. The subplots indicate: (A) accuracy of combined Hosts’ and automated annotations, and (B) accuracy of combined Visitors’ and automated annotations, both compared against the Archived annotations. The surfaces in (B) indicate the maximum and minimum combined accuracy among the five Visitors, and the solid lines indicate the mean. The black x on each curve indicates the point where *κ*_coral_ was 5% lower than its maximum value (i.e. a 5% drop compared to the *κ*_coral_ of the (A) Hosts, and (B) the mean of the Visitors).

Alleviate is a semi-automated annotation mode, in which the automated annotator has the option of deferring difficult decisions to a human expert annotator. The ratio of points that are automatically classified was denoted ‘level of alleviation’, *λ*. Because the expert annotators were more accurate than the automated annotator, the accuracy of Alleviate increased as *λ* decreased, and the highest accuracies were observed when *λ* = 0%, i.e. when all annotations were done by the Hosts (Fig 2). However, the trade-off curves between accuracy and automation allow for large alleviation with sustained high accuracy (Fig 4). Specifically, using the full training-set of available expert-annotated images in the present study, *λ* = 38–55% incurred < 5% decrease in *κ*_coral_ compared to the Hosts’ annotations (Fig 4A). Similarly, when alleviating the Visitors’ annotations, *λ* = 38–65% incurred < 5% decrease in *κ*_coral_ (Fig 4B). Moreover, a supplementary analysis established that *λ* = 32–45% incurred < 5% decrease in accuracy when only 5,000 manual point-annotations were used for training, which corresponds to ~ 25–200 training images for each of the four locations (S1 Appendix).

**Fig 5:**
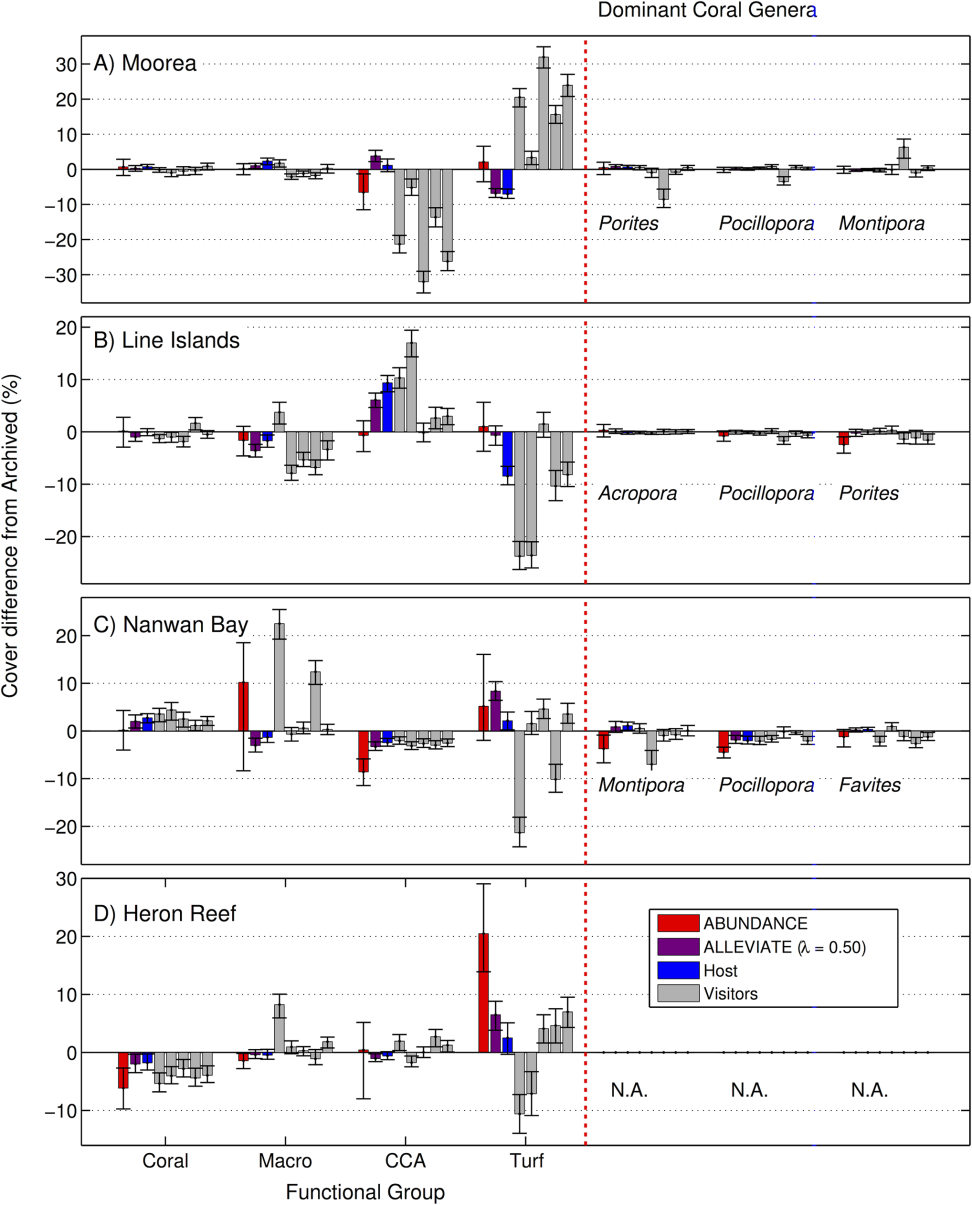
Percent cover estimation differences. Differences in percent cover estimates between the Host and Visiting experts as well as the Abundance and Alleviate operational modes and the Archived annotations. Differences are displayed as mean with 95% confidence interval (n = 200 images) for functional groups (coral, macroalgae, CCA, and turf) and the three most abundant coral genera in each location: (A) Moorea, (B) the northern Line Islands, (C) Nanwan Bay, and (D) Heron Reef. Archived describes reference annotations, performed by a local expert in each location, against which the other results were evaluated. Abundance and Alleviate refer to fully and semi-automated annotation modes. Host refers to re-annotation by the same local expert. Visitors refer to annotations completed by five coral biology experts who do not regularly work in those locations. Genus-level identification for Heron Reef is not provided, as Archived annotations were not available to this resolution.

**Fig 6:**
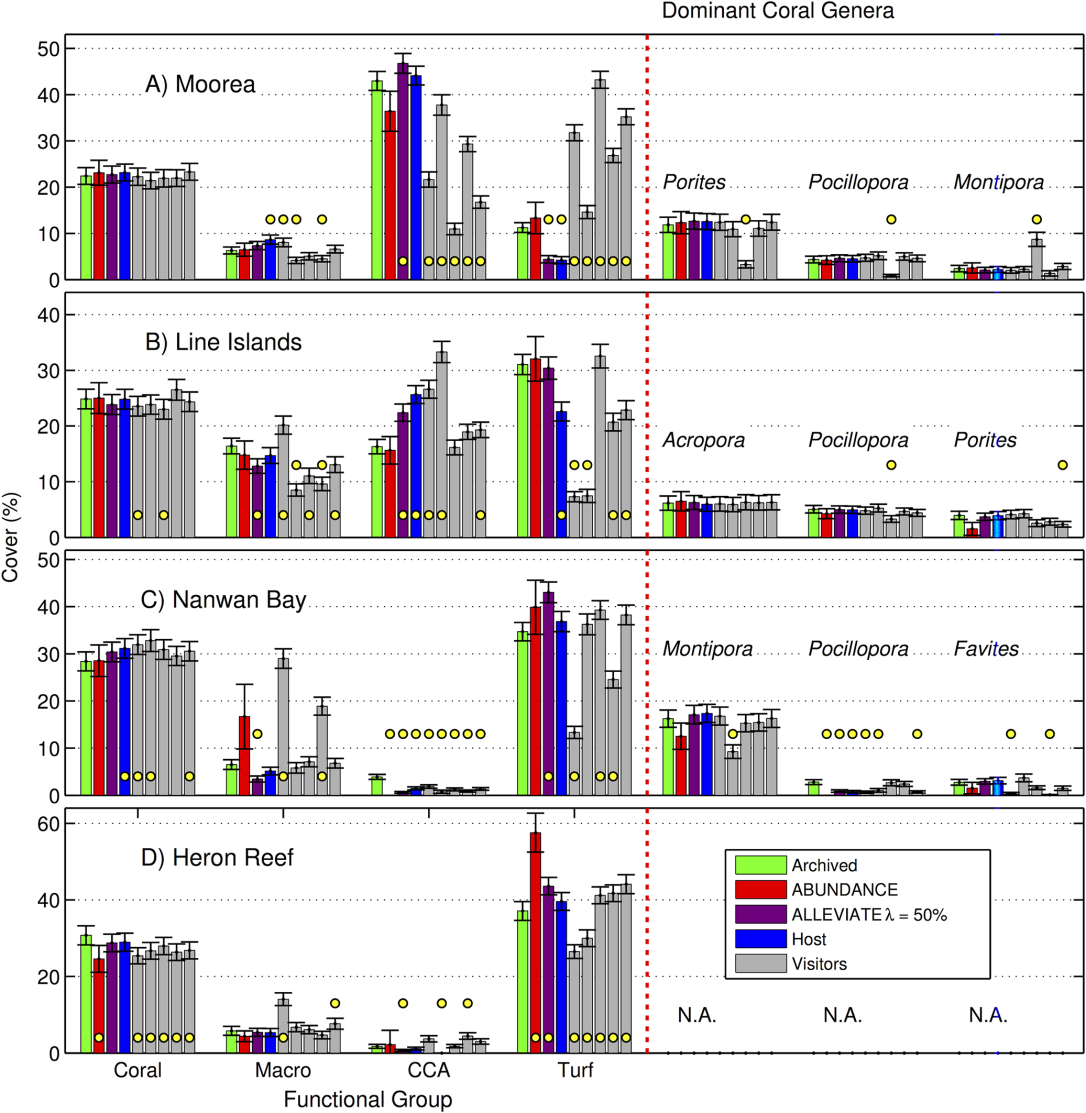
Percent cover estimates. Percentage cover estimates of functional groups (coral, macroalgae, CCA, and turf algae) and the three most abundant coral genera in each location as determined by different annotation methods. Values displayed as mean ± SE (n = 200 images) for each location: (A) Moorea, (B) the northern Line Islands, (C) Nanwan Bay, and (D) Heron Reef. Archived describes reference annotation, performed by a local expert in each location, against which the other results were evaluated. Abundance and Alleviate refer to fully and semi-automated annotation modes. Host refers to re-annotation by the same local expert. Visitors refer to annotations completed by five coral ecology experts who do not regularly work in the locations. Yellow dots indicate significant differences at 95% confidence between cover estimates from Archived versus other annotations for that label and dataset. Genus-level identification for Heron Reef is not provided, as Archived annotations were not available to this resolution.

The Mean Average Errors (MAEs) of cover estimates obtained using Alleviate at *λ* = 50%, compared to cover estimates from the Archived annotations, were 1.3 ± 0.4% for coral, 2.0 ± 0.8% for macroalgae, 3.6 ± 1.0% for CCA, and 5.6 ± 1.7% for turf algae (mean ± SE, n = 4 locations, Fig 5). The 1.3% coral cover MAE corresponds to a relative error of ~ 4–6% at the 22–31% coral cover recorded across the four locations (Fig 6). These results should be viewed in the context of the Hosts’ MAEs, which were 1.3 ± 0.6% for coral, 1.5 ± 0.4% for macroalgae, 3.4 ± 2.0% for CCA, and 5.0 ± 1.6% for turf algae (mean ± SE, n = 4, Fig 5); and the Visitors’ MAEs, which were 2.2 ± 0.4% for coral, 4.2 ± 1.2% for macroalgae, 7.6 ± 2.1% for CCA, and 11.9 ± 2.0% for turf algae (mean ± SE, n = 20, Fig 5). The Alleviate MAE for the three dominant coral genera found in Moorea, the Line Islands, and Nanwan Bay was 0.5 ± 0.2% (n = 9, Fig 5). Again, this was similar to the Hosts’ MAE of 0.5 ± 0.2% (n = 9, Fig 5), and lower than the Visitors’ MAE of 1.3 ± 0.3% (n = 45, Fig 5). Notably, the Alleviate cover estimates for the three dominant coral genera in each location was only different from the Archived cover estimates for one genera: *Pocillopora* in Nanwan Bay (Fig 6, S2 Table). It was also not different from the Archived cover estimates with respect to coral cover as a functional group in any of the locations (Fig 6, S2 Table).

The difference in accuracy *κ* ^Alleviate^ – *κ* ^Host^ (at *λ* = 50%) was not correlated with benthic cover (r = 0.25, df = 33, p = 0.14, Fig 3B). The differences for coral as a group were around -5% for the four locations, and between -9% and +3% for the coral genera (a positive difference means that *κ* ^Alleviate^ was higher than *κ* ^Host^). The only exception was *Platygyra*, where the difference was -28.0% at 1.6% cover and -15.3% at 0.5% cover in Nanwan Bay and Line Islands respectively. The differences for algal functional groups were > -10%, except for macroalgae and CCA in Nanway Bay where the differences were -19.8% at 6.5% cover, and -15.6% at 3.9% cover, respectively.

#### Fully automated annotation using Abundance

Using the abundance correction method of [35], the Abundance operational mode can be deployed without any manual annotations. The MAE of cover estimates obtained in the Abundance mode was 1.8 ± 1.5% for coral, 3.4 ± 2.3 for macroalgae, 4.1 ± 2.1% for CCA, and 7.2 ± 4.5% for turf algae (mean ± SE, n = 4, Fig 5). The Abundance cover MAE of the three dominant coral genera of Moorea, the Line Islands, and Nanwan Bay was 1.5 ± 0.5 (n = 9, Fig 5). As with Alleviate, these errors should be viewed in the context of the human errors.

## Discussion

### Annotator Accuracy

We have estimated the inter- and intra- annotator variability of human experts for annotating coral reef survey images. Quantifying this variability is critical to contextualize the performance of automated annotation methods. For example, if the accuracy of human experts were low for a certain substratum, the automated annotation accuracy would be expected to be equally low (since automated annotation methods are commonly trained on archived, manually annotated data). Conversely, for labels where the human annotator accuracy is high (e.g., for coral genera), our results establish baselines against which newer generations of automated annotation systems can be compared.

#### Intra-annotator variability

Annotator accuracy was measured using Cohen’s kappa (*κ*) for intra- and inter- annotator agreement. While *κ* is widely used to assess annotator agreement for categorical data [38–40], there is no absolute scale against which values of *κ* can be gauged. For example, [41] characterized *κ* < 0 as indicating no agreement, 0–20% as slight agreement, 21–40% as fair agreement, 41–60% as moderate agreement, 61–80% as substantial agreement, and > 81% as almost perfect agreement. In contrast, [42] characterized *κ* < 40% as poor agreement, 40–75% as fair-to-good agreement, and > 75% as excellent agreement. Using these interpretations, the Host *κ*_coral_ of 89.7±1.2% should be considered an “excellent” or “almost perfect” agreement between the Host and Archived annotations. Similar high accuracies have previously been noted for self consistency of human annotations of corals versus other substratum [13,43]. Ninio et al. investigated accuracy (as defined by agreement with *in-situ* observations) for video transects from coral reefs, and observed 96% accuracy for identifying hard corals [13].

The intra-annotator accuracy of identifying algal substrata was lower than for corals. In particular, for CCA and turf algae Cohen’s kappa were 51.0±9.3% and 61.1±6.1%, respectively, although this agreement can still be considered ‘moderate’ or ‘fair to good’ [41,42]. The lower accuracy of algal classification may be due to methodological limitations in discriminating algae in planar RGB photo-quadrats at common resolutions as used in the present study (Table 1), but also due to the label definitions of the consensus label-set. For example, the consensus label-set defined turf-algae as a “multi-specific assemblage < 1 cm in height”, while macroalgae were “larger algae > 1 cm in height” (Table 2). Considering the planar nature of the photo quadrats, such distinctions can be hard to apply consistently across photographs. Ninio et al. observed 80% annotation accuracy of algae (as a ‘main benthic’ group) [13], which is higher than our estimates of algal functional groups annotation accuracy. However, comparison of accuracy of algae as a ‘main benthic’ group, with algal functional groups (macroalgae, turf algae, and CCA) is misleading, since the majority of the algal classification errors occurred among the algal groups (S3 Fig). Merging the algal functional groups used in this study, the Host accuracy of discriminating algae (macroalgae, turf algae and CCA combined) versus everything else, was *κ*_algae_ = 71.1±1.6% (mean ± SE, n = 4), which is similar to the 80% accuracy recorded in [13].

The Hosts’ accuracies were not correlated with the abundance of the substratum of interest, and were > 60% except for five rare substrata occupying < 5% of the benthos (Fig 3). The only additional exception was turf algae in Moorea, with 43% accuracy at 11.3% cover. These results agree with the findings of Ninio et al. who noted highly variable precision when mean covers were < 3%, but “markedly decreased” variability at higher covers (i.e., > 3%) [13].

#### Inter-annotator variability

Inter-annotator accuracy was lower than intra-annotator accuracy. Differences were large in particular for CCA and turf algae, where the accuracies were 35.5±3.7% and 43.3±3.6% respectively, which can be considered “fair” [41,42]. This lower accuracy resulted in cover estimation errors by the Visitors that commonly exceeded 10% (Fig 5). These results are in contrast with the results of Ninio et al., where the intra-annotator variability only contributed ± 0.5% to the confidence intervals for benthic group ‘Algae’ [13]. We believe this contrast has two principal causes. First, the multiple human annotators of Ninio et al. were all familiar and trained in the ecology of the study location [13], while the Visitors of the present study were less familiar with the local ecology and had not (with a few exceptions) been physically present at the respective locations. Second, as noted above, Ninio et al. do not report cover estimation on algal functional groups but only in aggregate. Still, it is clear from our results that rigorous training of expert annotators is critical to achieve reliable manual annotation of turf and CCA algal groups, in particular where there are multiple experts involved in the annotation process. Another alternative is to use complementary imaging techniques, such as fluorescence photography [44,45], to make the annotation task less ambigious.

### Modes of Automated Annotation

Our results indicate that an automated annotator, deployed in our semi-automated annotation mode, Alleviate, can make 50% of the annotation decisions without affecting the quality of the percent cover estimates (Figs 5 and 6), even when limited data are available for training the automated annotator (S1 Appendix). In particular, Alleviate cover estimates for the three dominant coral genera in each location, and for the coral functional group, were not different from the Archived cover estimates (Fig 6). The only exception was *Pocillopora* in Nanway Bay which may be due to an erroneous Archived cover estimate, since also the Host’s, and three of the Visitors’, cover estimates differed (Fig 6). Since the investigated locations exhibit a wide variety of photographic methodology and habitat structures (Table 1), we believe these results generalize to other coral reef survey locations, suggesting a wide applicability of Alleviate to reduce manual annotation work. We further believe that the deployment of Alleviate may increase the accuracy of the human annotators by allowing them to focus their attention on a subset of the annotation decisions. This effect has been demonstrated for semi-automated annotation of plankton samples [46], but would need to be verified in a future user-study for the present application.

Differences in accuracy between Alleviate and the Hosts were generally small (< 10%), and not correlated with the substratum abundances (Fig 3). Two outliers were noted. First, the accuracy of Alleviate for *Platygyra* was 15–29% lower than for the Hosts (recorded at < 2% cover). This may be due to the limited amount of available training data for *Platygyra* (due to the low cover), and the visual similarity to other massive or encrusting corals with meandroid or submeandroid corallum morphologies. Second, the accuracy of Alleviate was 16% and 20% lower for macroalgae and CCA in Nanwan Bay respectively (both recorded at < 7% cover). This may be due to a combination of limited amount of available training data, and limitations in the photographic methodology indicated by the low accuracy of the Host: 60.4% and 30.3% respectively for macroalgae and CCA (Fig 2, S1 Table).

Alleviate uses the classification scores to decide when to make an automated annotation and when to defer to a human expert. To the best of our knowledge, this approach has not ben utilized for annotation of coral reef survey images. However, a similar approach was used for classification of plankton images acquired using imaging in-flow cytometry, where the specificity of the classifier increased for all classes when only considering decisions where the classifier was > 65% confident [47,48].

We have also evaluated the efficacy of a fully automated annotation mode Abundance. Our results indicates that Abundance generates unbiased cover estimates, but with larger standard errors (Fig 5), which is to be expected by the design of the abundance correction method [35]. Large standard errors are undesirable, but can be reduced by collecting and automatically analyzing more images. In fact, the introduction of fully automated annotation can inform the underlying random sampling survey design, where for example, more images of coral reefs could be collected in the field (which is often relatively easy and cost effective) in order to compensate for the larger variance of fully automated annotation modes such as Abundance [ 49 ].

As with Alleviate, the underlying principle of Abundance has been utilized in other applications. Indeed, it was originally proposed for automated plankton classification [35], and it has been commonly utilized for that purpose [47]. It has also been utilized for sentiment analysis from text corpora [36].

In order for Abundance to generate unbiased cover estimates, the confusion matrix for the data that is being sampled must be known [35]. In our experiments, the training data (i.e., the Reference Sets) were drawn from the same underlying probability density as the sampled data (i.e., the Evaluation Sets), and the confusion matrices could thus be estimated from the training data. If, however, the training data and the sampled data are drawn from different locations, sites, or years, the probability densities may be different [50,51]. In such situations a subset of the sampled data must be annotated in order to estimate the confusion matrix, and Abundance can no longer be considered fully automated. However, it may still offer significant savings compared to Alleviate or fully manual annotation.

## Conclusions

We have established baseline accuracies of human expert annotations of coral reef survey images, and compared them to a recent method for automated annotation. Our results indicate that the accuracy of human experts varies with the type of benthic substrata. Annotations of coral genera have low inter- and intra- annotator variability, while annotations of algal groups, in particular turf and CCA algae, from those same survey images, have much larger intra- and inter- annotator variability. This suggests the need for development of photographic methodology for visualizing turf and CCA algal groups, and for rigorous training of expert annotators.

We have proposed two modes of operations in which methods for automated annotation can be deployed semi- or fully- automated. Our results indicate that cover estimates from the semi- automated annotation mode, Alleviate are of similar quality to cover estimates of manual annotations, while the cover estimates of the fully- automated mode, Abundance, are unbiased but with higher variance. The appropriate deployment mode will depend on the specific application. A reef manager for example, might utilize Abundance for rapid assessment of reef health, while a benthic ecologist might prefer Alleviate, which requires greater manual effort but is more accurate. We expect the reduction of annotation time to continue as improved methods for automated annotation become available. Implementations of the proposed modes of operation (Alleviate, Abundance, and Refine ( S1 Appendix )) are available to the public on CoralNet (coralnet.ucsd.edu).

## Supporting Information

S1 Appendix Supplementary analysis and information. Content: (1) Details of Coral Reef Survey Locations; (2) Classification Using Linear Support Vector machines; (3) Importance of Training Size for Alleviate; (4) Refine: a Supplementary Operational Mode. S1 Appendix also includes figures related to the appendix.
(PDF)

S1 Fig Sample photoquadrats. Sample photoquadrats drawn from the long-term coral reef projects that served as test data for the present analysis. (First row) 4 photoquadrats (50 × 50 cm) from Moorea; (second row) 3 photoquadrats (65 × 90 cm) from the Line Islands; (third row) 4 photoquadrats (35 × 35 cm) from Nanwan Bay (Taiwan); (bottom row) 3 photoquadrats (50 x 65 cm) from Heron Reef (GBR).
(PDF)

S2 Fig Screenshots from CoralNet. A) Graphical user interface used to create the Hosts’ and Visitors’ annotations. B) Browse tool used by the Visitors to learn about the images and the label-set from previous annotations. The screen-shot shows the result of a user searching for all *Pocillopora* annotations from the Line Islands dataset.
(PDF)

S3 Fig Confusion matrices. Confusion matrices for Moorea, Line Islands, Nanwan Bay and Heron Reef. Values at row *r*, column *c* in the matrices indicate the ratio of annotations originally labeled by Archived as label *r* now classified by the Host, Visitors, and automated annotator, respectively as label *c*. The numbers on the right margin indicate the total count of each row. For brevity, all annotations of the Visitors are merged into a single confusion matrix and only labels for which more than 10 annotations were assigned by any of the annotators were included.
(PDF)

S1 Table Tabulated accuracies. Annotation accuracies as measured by Cohen’s kappa for the automated annotator (Aut.), Alleviate at *λ* = 50% (ALL.), Hosts, and Visitors. All accuracies are measured as compared to the Archived annotations. The first row in each location is the accuracy of the full confusion matrix, while the other rows indicate the accuracy of binary classification between the indicated label or label group and the other labels. These labels or label groups are: functional groups coral, macroalgae, crustose coralline algae (CCA), and turf algae, followed by the dominant coral genera (i.e. with > 10 Archived annotations), and the hydrozoan *Millepora* if present in that location. The coral genera are ordered by percent cover based on the Archived annotations. Note that coral genera are not included for Heron Reef because the annotations were not resolved to genus level in the original study. The rightmost column shows the percent cover based on the Archived (Arch.) annotations.
(PDF)

S2 Table Tabulated p-values. Probabilities that the estimated cover from a set of annotations and for a certain label and location is the same as the cover estimated from the Archived annotations. Probabilities (p-values as estimated from the permutation t-test**)** in red italics are below 0.05 / 8 = 0.00625, which means that the null hypothesis can be rejected at a 95% confidence level with a Bonferroni correction for eight repeated measurements. This implies that the particular set of annotations is unreliable for cover estimation. For each location, the first four rows are the functional groups: coral, macroalgae, crustose coralline algae (CCA), and turf algae, followed by the dominant coral genera (i.e. with > 10 Archived annotations), and the hydrozoan *Millepora* if present in that location. The coral genera are ordered by percent cover based on the Archived annotations. Note that coral genera are not included for Heron Reef because the annotations were not resolved to genus level in the original study. Columns ABU and ALL are the Abundance and Alleviate annotation modes respectively, and V1 to V5 are the Visitors. The rightmost column shows the percent cover based on the Archived (Arch.) annotations.
(PDF)
