# Supplementary material for: Towards Automated Annotation of Benthic Survey Images: Variability of Human Experts and Operational Modes of Automation
Source: PLoS One. 2015 Jul 8;10(7):e0130312. doi: 10.1371/journal.pone.0130312 (PMC4496057; doi:10.1371/journal.pone.0130312)
Supplement: S1 Appendix — Content: (1) Details of Coral Reef Survey Locations; (2) Classification Using Linear Support Vector machines; (3) Importance of Training Size for Alleviate; (4) Refine: a Supplementary Operational Mode. S1 Appendix also includes figures related to the appendix. (PDF) [file pone.0130312.s001.pdf]

S1 Appendix: Towards automated annotation of benthic survey images: variability of human experts and operational modes of automation

DETAILS OF CORAL REEF SURVEY LOCATIONS 2

MOOREA 2

NORTHERN LINE ISLANDS 2

NANWAN BAY, TAIWAN 3

HERON REEF, GREAT BARRIER REEF 3

CLASSIFICATION USING LINEAR SUPPORT VECTOR MACHINES 5

IMPORTANCE OF TRAINING SIZE FOR ALLEVIATE 6

REFINE: A SUPPLEMENTARY OPERATION MODE 8

REFERENCES 10

Details of coral reef survey locations

The acquisition and analysis methods used in the coral surveys, which provided images and Archived annotations for the present study, are detailed in this section.

Moorea

In Moorea, sampling took place in fringing (2–5 m depth) and outer (10-m and 17-m depth) reef habitats, with two sites on each of the three shores of the island [1]. In each habitat, images (each 0.25 m^2) were recorded at ~ 40 points scattered randomly along a 40 m transect using a Nikon D70 camera (6.24 megapixels) attached to two strobes and mounted to a frame holding the camera perpendicular to the reef. With this sampling scheme, ~ 720 images were recorded, and the resolution of the camera allowed objects as small as ~ 1 cm to be resolved. Images were analyzed using Coral Point Count with Excel Extensions (CPCe) software [2], in which a grid of 200 randomly-located dots was superimposed on each image, and the taxon beneath the dots identified by a human annotator. The organisms covering the coral reefs were resolved to four functional groups: scleractinian corals, macroalgae [algae ≥ 1 cm high], algal turf [algae < 1 cm high], and crustose coralline algae (CCA), with the scleractinians further resolved to genus.

Northern Line Islands

Images from the Line Islands were recorded on outer reef communities (10-12 m depth) at Kingman, Palmyra, Tabuaeran and Kiritimati atolls in 2005. On each island, ten sites were surveyed with ~ 200 images per site, and at each site two 25 m transects were

deployed with 10 randomly placed photoquadrats (0.54 m^2) along each transect. No strobes were used, and the white balance of the camera was set prior to shooting using a white reference. Images were recorded using an Olympus C-7070 camera (7.1 megapixels) in an underwater housing, and were edited for brightness and contrast in Photoshop. Benthic analysis was performed using PhotoGrid 1.0 with 100 points per photograph. Organisms were identified to genus when possible, but genus-level annotations were pooled to six groups: scleractinian coral, soft coral, macroalgae, CCA, turf algae, and "other".

Nanwan Bay, Taiwan

A total of 890 images were recorded in southern Taiwan at two sites in Nanway Bay (Houbihu and Outlet) on 3 occasions during 2011 and 2012, and at 5 sites around Liuchiu Island during 2007 and 2011. At each site (all 2–5 m depth), ~ 30 photoquadrats (0.123 m^2) were recorded at random locations along three 10 m transects [3]. Images were recorded with a Canon G12 camera (9.98 megapixels) in an underwater housing mounted to a frame holding the camera perpendicular to the reef. The percentage cover of each benthic category was determined using CPCe software with 50 points distributed randomly on each photo and then assigned to 154 benthic cover types [2].

Heron Reef, Great Barrier Reef

Heron Reef images were recorded coinciding with satellite imagery as part of an annual mapping survey in 2007 [4]. Geo-referenced photoquadrats (1 m^2) were recorded at ~ 3m intervals along transects using a Canon A540 camera (6.2 megapixels) held 0.5 m above

the benthos. The position of each photoquadrat was geo-referenced using a GPS floating at the surface and towed behind the operator. Thirty-two transects were censused, each between 200 and 1000 m in length for a total of ~ 13 km (3,500 photos). The benthic cover category for each photo was determined by randomly distributing 24 points on each photograph, then manually assigning each point to one of 92 benthic cover types using CPCe [2]. The coral categories were resolved to functional group (e.g. branching coral) instead of genus as in the other three locations.

Classification using linear support vector machines

A binary linear Support Vector Machine [5] solves the following unconstrained optimization problem,

$$\min_{\omega}\left[\frac{1}{2}\omega^T\omega + C\sum_{i=1}^{n}\xi(\omega;\ x_i, z_i)\right]$$

where $z_i \in \{-1, 1\}$ is a binary label, x_i is a data sample, $C > 0$ is a regularization parameter and $\xi(\omega; x_i, y_i) = \max(1 - z_i\omega^T x_i, 0)$ is a loss function [6]. The weight vector $\omega \in \mathbb{R}^d$ is the output of the learning algorithm and is used to classify new, unseen sample, x_u as

$$z_u = \mathrm{sign}(\omega^T x_u)$$

Multiclass classification can be mapped to binary classification through a one-versus-rest classification scheme. In this scheme a weight vector ω_m is trained separately for each class m, by converting the multiclass labels $y_i \in \{1, \dots, 20\}$ to binary labels

$$z_i = \mathbb{S}(y_i, m), i \in 1, \dots, n$$

where $\mathbb{S}$ is the signed indicator function

$$\mathbb{S}(a, b) = \begin{cases} 1\ if\ a = b \\ -1\ if\ a \neq b \end{cases}$$

Classification is then done by selecting the class for which the score $s_u(m) = \omega_m^T x_u$, has the highest value

$$y_u = \mathrm{argmax}_m[s_u(m)]$$

Importance of training size for ALLEVIATE

In this analysis the accuracy of ALLEVIATE, in terms of κ_{coral}, was measured as a function of training data size. (As defined previously, κ_{coral} is the Cohen's Kappa [7] score of classifying coral versus non-corals.) As detailed in the paper, after randomly selecting the 200 images in the Evaluation Set, the remaining data was referred to as the Reference Set, and used to train the machine-learning algorithm. The total number of training samples available for each location is detailed in Table 1, and varied between 34,260 and 94,200. In 30 repeated experiments, a random subset of increasing size was selected from this training data, so that *e.g.* $94{,}200 \times \frac{i}{30}$ samples were used in iteration i for Moorea. This data was used to train an automated annotator, and generate automated annotations for the Evaluation Set. These annotations, together with the Host annotations were used to calculate κ_{coral} for 30 levels of alleviation ($\lambda = 0, \ldots, 100\%$). This procedure was repeated 30 times for each location, and the mean results for each training set size and level of alleviation is shown in Figure A1. The results are normalized so that κ_{coral} for the Host annotator of each location is 1. The level curves thus indicate what proportion of points can be classified by the automated annotator while maintaining a certain accuracy ratio compared to the Host. For example, with a 5% decrease in κ_{coral}, ~10,000 training samples are required in Moorea for 50% alleviation, and ~90,000 for 60% alleviation. The left side of Figure A1 shows that the accuracy of the automated annotations increases as more training samples become available, but that the increase-rate decreases as additional training samples are added. On the right side of Figure A1 are the 95%-level curves for each location plotted on the same axis for comparison. The

lower automated annotation performance on Nanwan Bay and Heron Reef compared to Line Islands and Moorea, may be due to the difference in photographic quality between the locations. This analysis indicates that automated annotation performance saturates around 50,000 training samples. For this amount of training data, 45–60% alleviation results in 95% of max κ_{coral}, and 55-75% alleviation result in 90% of max κ_{coral}.

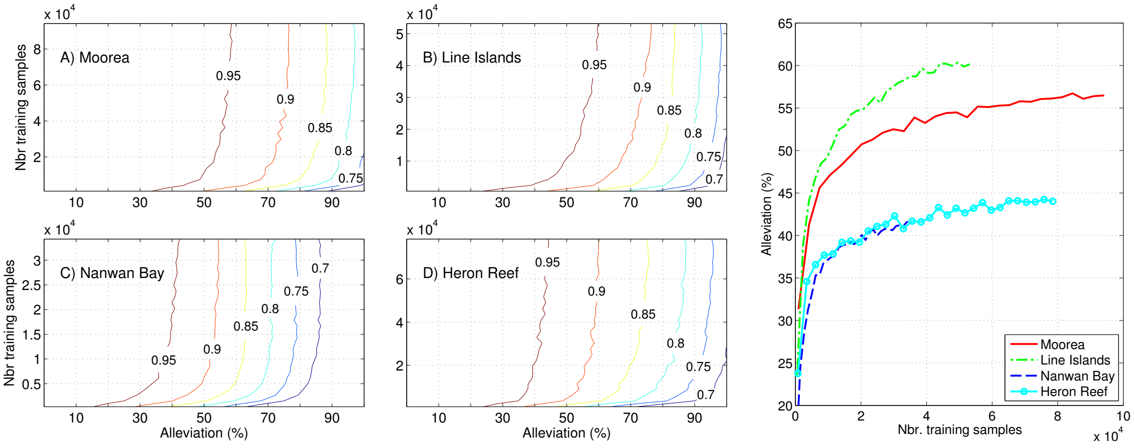

Figure A1: Training set size analysis. Left: level curves indicating the accuracy of ALLEVIATE at various levels of alleviation and training set sizes. Accuracy is expressed as the normalized κ_{coral} score (so that κ_{coral} at $\lambda = 0\%$ alleviation is 1). Right: 95% level curves for the four locations drawn on the same axis. Note how the better quality photographs of Moorea and Line Islands enable stronger scores for the same amount of training data compared to Nanwan Bay and Heron Reef. Also note how the main gain in alleviation level is achieved, in general, by the first 50,000 training samples.

REFINE: a supplementary operation mode

Label-sets of benthic surveys can include over 100 labels when fine taxonomic resolution is required. In such situations, point annotation tools such as CPCe [2] suffer due to limited visual resolution and tedious manual distinction among categories. We define an interactive annotation mode, REFINE, that addresses this problem. As denoted in the paper, $s_{i,k}(m)$ is the score given to class m by the automated classification algorithm for a certain image i and point k; these scores are used also by REFINE. A set of t labels (where t is smaller than the total number of labels) with the highest scores is retrieved from the full label-set and displayed to the human annotator. The human annotator selects the correct label from the retrieved set, or asks to see more labels. This allows the annotator to quickly identify the correct label, and avoids the problems associated with limited screen-space. In the case of $t = 1$, the annotator can rapidly verify if the sole label is correct, or else select from the full label-set.

We conducted an experiment to measure to what extent the retrieved set of REFINE contains the correct label. For each point in each image, the t labels with highest scores were retrieved. If the Host annotation for that point was among the retrieved labels, it was assigned to that point; otherwise the retrieved label with the highest score was assigned. The full set of annotations thus derived were compared to the Archived annotation to calculate Cohen's kappa (κ) [7]. This procedure was repeated for each t until all labels were in the retrieved set, at which point $\kappa \approx 70\%$. The κ scores are plotted against t in Figure A2. The retrieval set sizes required for a 5% drop in κ compared to the Hosts were 5 Moorea, 7 for the Line Islands, and 16 for Nanwan Bay.

This indicates this strong ability of REFINE include the correct labels in the retrieved set. Since the label-set used for Heron Reef only contain 5 labels, it was not included in this experiment.

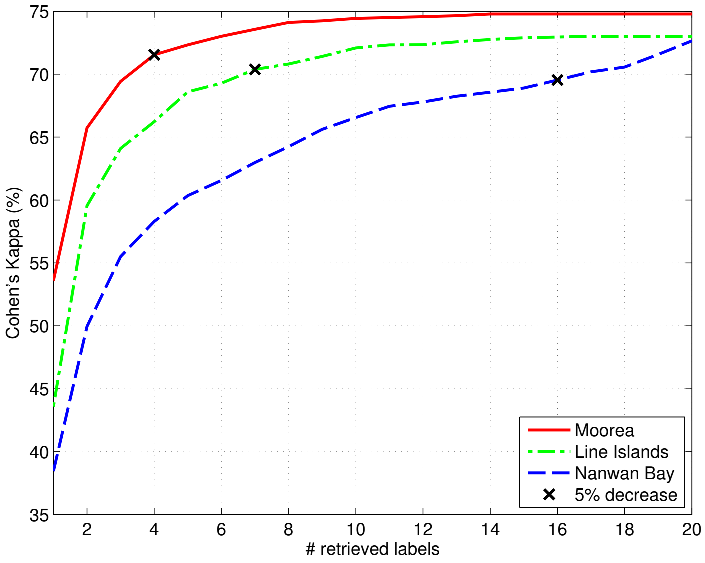

Figure A2: Cohen's kappa (κ) for various size retrieval sets of REFINE for Moorea, Line Islands and Nanwan Bay. The κ was calculated by comparing the joint set of Host and automated annotations to the Archived annotations. The black x on each curve indicates the point where κ is 5% lower than its maximum value (i.e. a 5% drop compared to the κ of the Hosts). Note how the better automated annotation accuracy for Moorea and Line Islands enable smaller sets of labels to be retrieved compared to Nanwan Bay.

References

1. Adam TC, Schmitt RJ, Holbrook SJ, Brooks AJ, Edmunds PJ, Carpenter RC, et al. Herbivory, connectivity, and ecosystem resilience: response of a coral reef to a large-scale perturbation. PLoS ONE. 2011;6: e23717.

2. Kohler KE, Gill SM. Coral Point Count with Excel extensions (CPCe): A Visual Basic program for the determination of coral and substrate coverage using random point count methodology. Comput Geosci. 2006;32: 1259–1269.

3. Tkachenko KS, Wu B-J, Fang L-S, Fan T-Y. Dynamics of a coral reef community after mass mortality of branching Acropora corals and an outbreak of anemones. Mar Biol. 2007;151: 185–194.

4. Phinn SR, Roelfsema CM, Mumby PJ. Multi-scale, object-based image analysis for mapping geomorphic and ecological zones on coral reefs. Int J Remote Sens. 2012;33: 3768–3797.

5. Vapnik VN. Statistical learning theory. Wiley; 1998.

6. Fan R-E, Chang K-W, Hsieh C-J, Wang X-R, Lin C-J. LIBLINEAR: A library for large linear classification. J Mach Learn Res. 2008;9: 1871–1874.

7. Carletta J. Assessing agreement on classification tasks: the kappa statistic. Comput Linguist. 1996;22: 249–254.
