# Supplementary figures and images for: Towards Automated Annotation of Benthic Survey Images: Variability of Human Experts and Operational Modes of Automation
Source: PLoS One. 2015 Jul 8;10(7):e0130312. doi: 10.1371/journal.pone.0130312 (PMC4496057; doi:10.1371/journal.pone.0130312)

Moorea

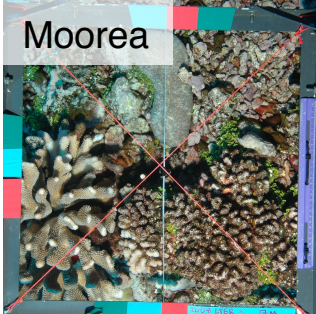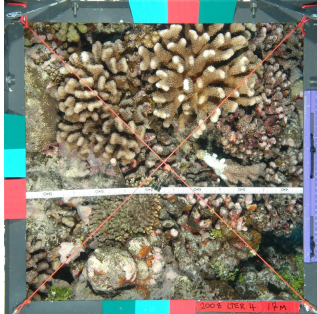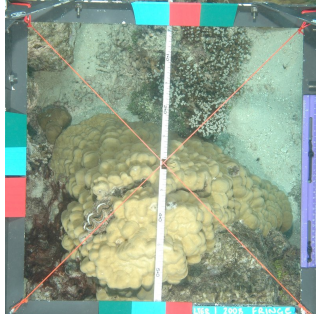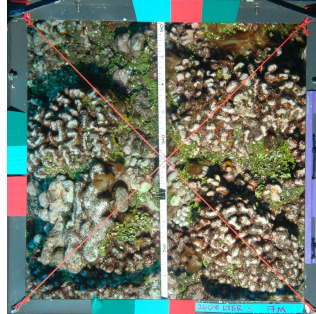

Line-Islands

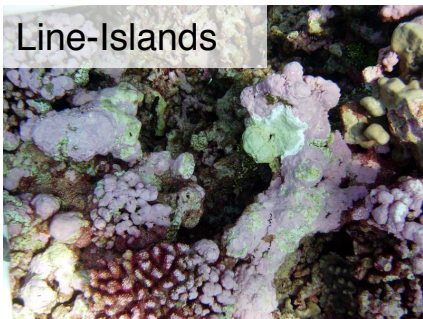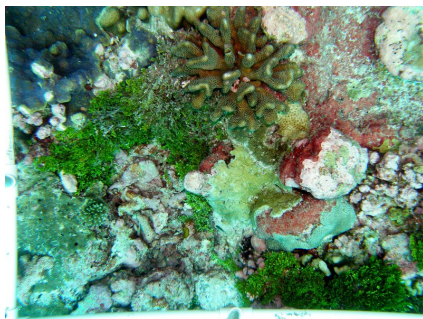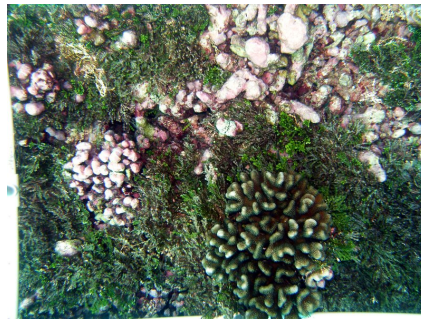

Nanwan Bay

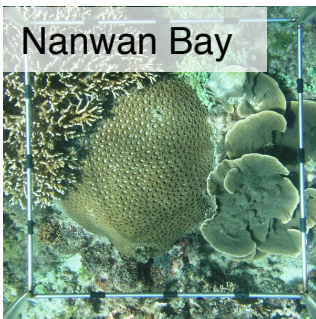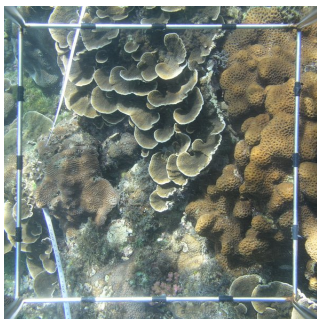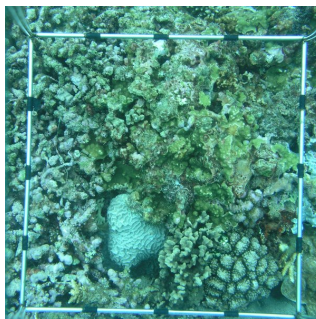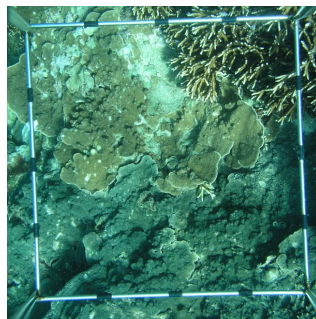

Heron Reef

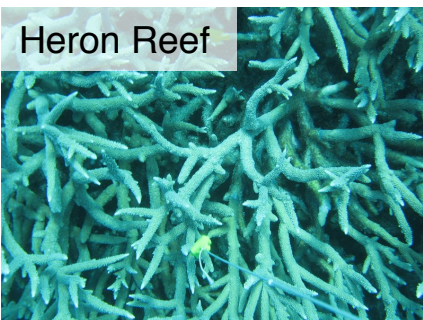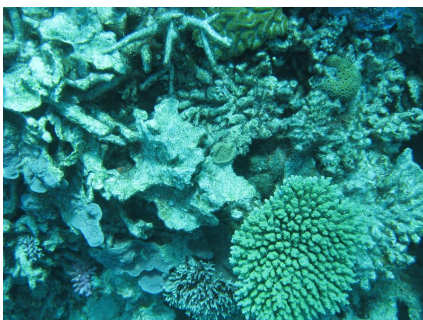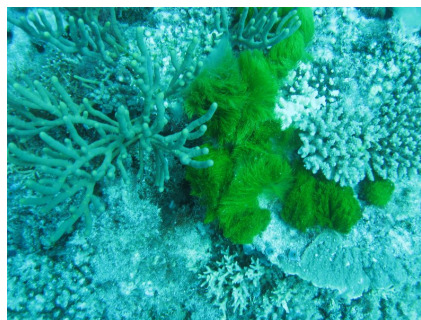

Supplement: S1 Fig — Sample photoquadrats drawn from the long-term coral reef projects that served as test data for the present analysis. (First row) 4 photoquadrats (50 × 50 cm) from Moorea; (second row) 3 photoquadrats (65 × 90 cm) from the Line Islands; (third row) 4 photoquadrats (35 × 35 cm) from Nanwan Bay (Taiwan); (bottom row) 3 photoquadrats (50 x 65 cm) from Heron Reef (GBR). (PDF) [file pone.0130312.s002.pdf]

## Moorea

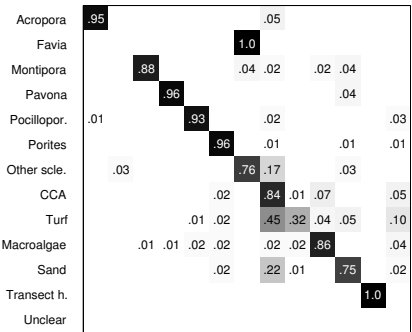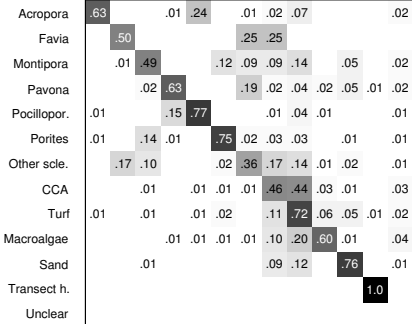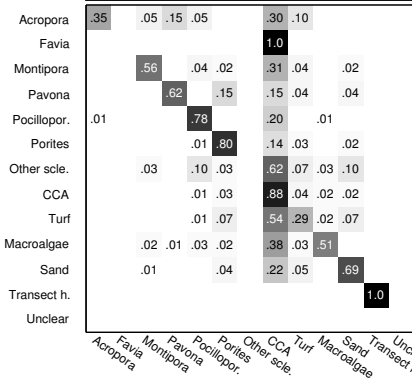

## Line Islands

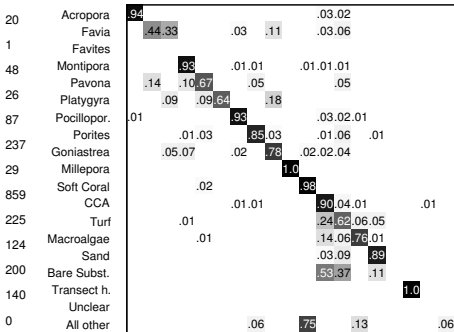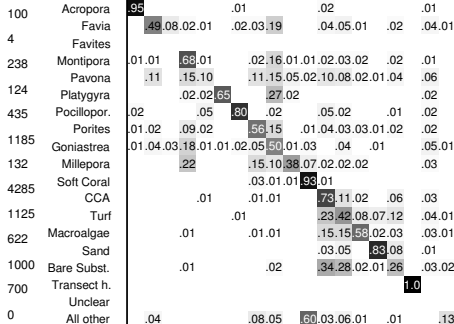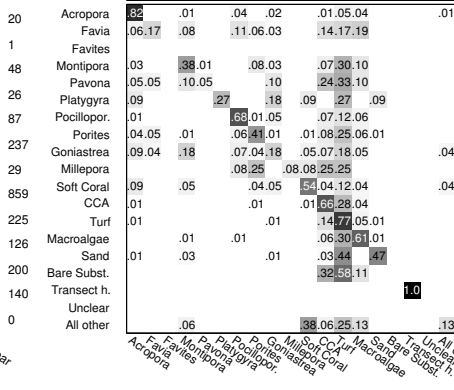

## Nanwan Bay

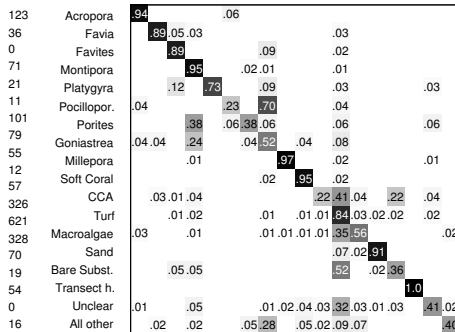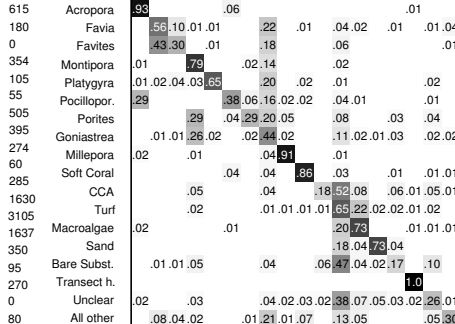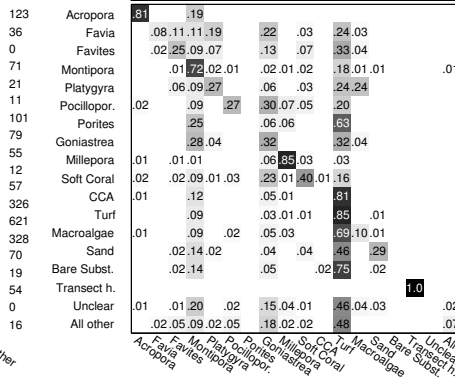

## Heron Reef

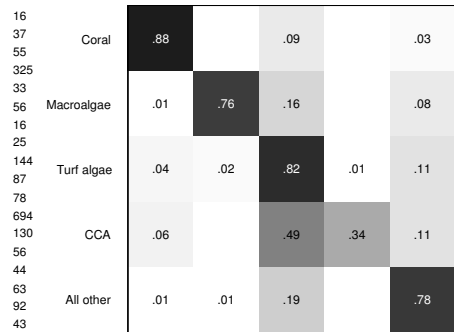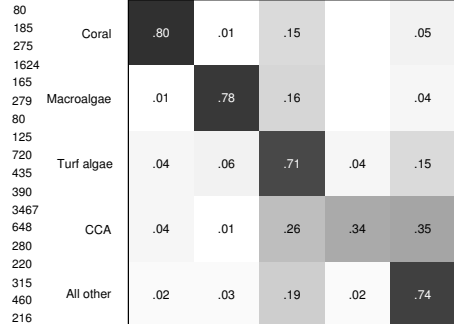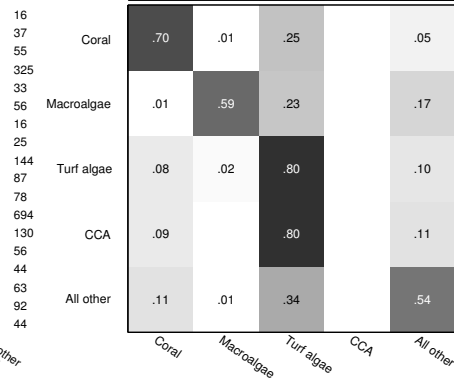

Host

## Visitors

## Automated

Supplement: S3 Fig — Confusion matrices for Moorea, Line Islands, Nanwan Bay and Heron Reef. Values at row r, column c in the matrices indicate the ratio of annotations originally labeled by Archived as label r now classified by the Host, Visitors, and automated annotator, respectively as label c. The numbers on the right margin indicate the total count of each row. For brevity, all annotations of the Visitors are merged into a single confusion matrix and only labels for which more than 10 annotations were assigned by any of the annotators were included. (PDF) [file pone.0130312.s004.pdf]
