# Supplementary material for: Towards Automated Annotation of Benthic Survey Images: Variability of Human Experts and Operational Modes of Automation
Source: PLoS One. 2015 Jul 8;10(7):e0130312. doi: 10.1371/journal.pone.0130312 (PMC4496057; doi:10.1371/journal.pone.0130312)
Supplement: S2 Fig — A) Graphical user interface used to create the Hosts’ and Visitors’ annotations. B) Browse tool used by the Visitors to learn about the images and the label-set from previous annotations. The screen-shot shows the result of a user searching for all Pocillopora annotations from the Line Islands dataset. (PDF) [file pone.0130312.s003.pdf]

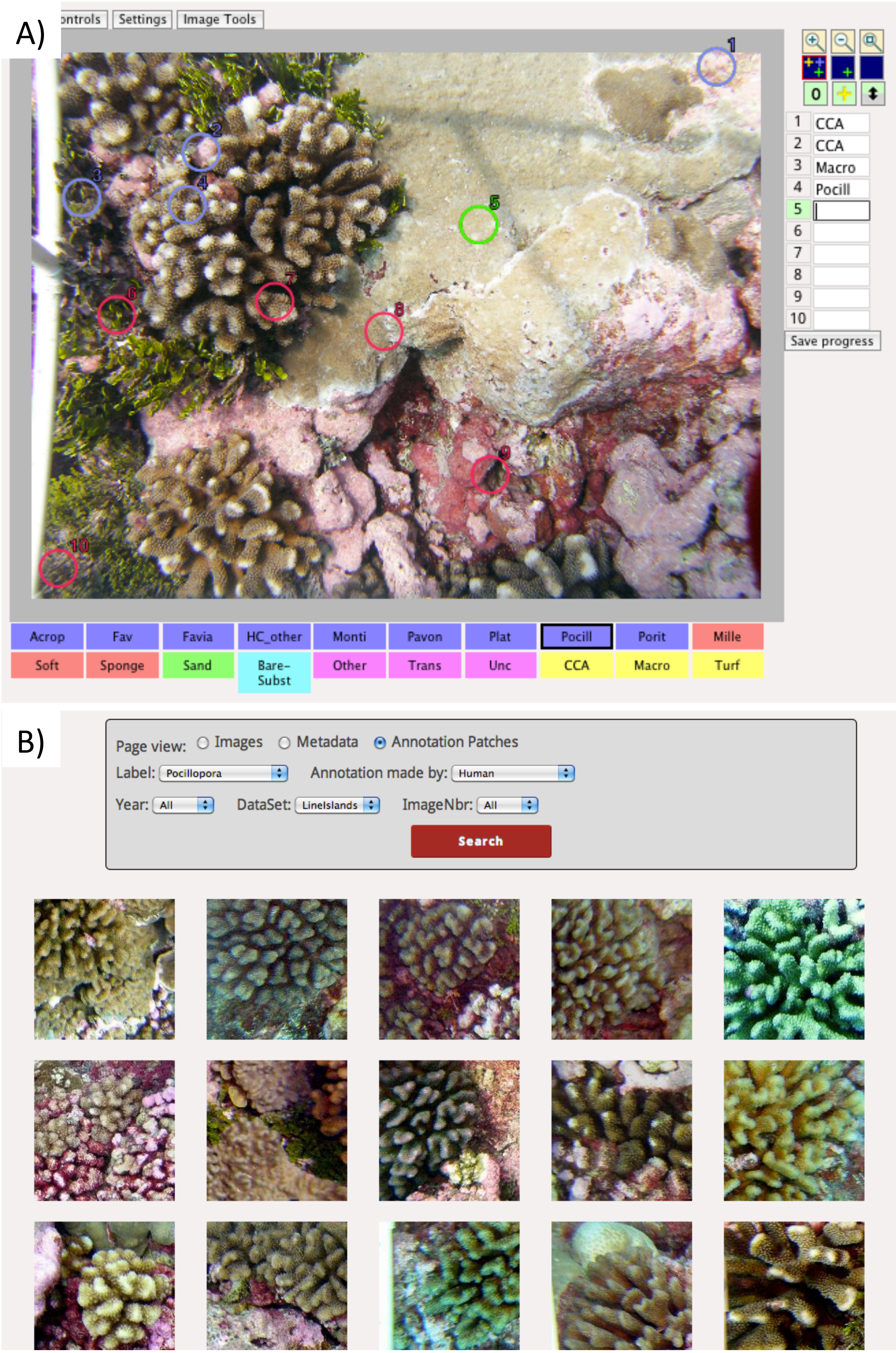
A)
ontrols
Settings
Image Tools
1 CCA
2 CCA
3 Macro
4 Pocill
5
6
7
8
9
10
Save progress
Acrop
Fav
Favia
HC_other
Monti
Pavon
Plat
Pocill
Porit
Mille
Soft
Sponge
Sand
Bare-Subst
Other
Trans
Unc
CCA
Macro
Turf
B)
Page view: Images Metadata Annotation Patches
Label: Pocillopora
Annotation made by: Human
Year: All
DataSet: LineIslands
ImageNbr: All
Search
