# Supplementary material for: Towards Automated Annotation of Benthic Survey Images: Variability of Human Experts and Operational Modes of Automation
Source: PLoS One. 2015 Jul 8;10(7):e0130312. doi: 10.1371/journal.pone.0130312 (PMC4496057; doi:10.1371/journal.pone.0130312)
Supplement: S1 Table — Annotation accuracies as measured by Cohen’s kappa for the automated annotator (Aut.), Alleviate at λ = 50% (ALL.), Hosts, and Visitors. All accuracies are measured as compared to the Archived annotations. The first row in each location is the accuracy of the full confusion matrix, while the other rows indicate the accuracy of binary classification between the indicated label or label group and the other labels. These labels or label groups are: functional groups coral, macroalgae, crustose coralline algae (CCA), and turf algae, followed by the dominant coral genera (i.e. with > 10 Archived annotations), and the hydrozoan Millepora if present in that location. The coral genera are ordered by percent cover based on the Archived annotations. Note that coral genera are not included for Heron Reef because the annotations were not resolved to genus level in the original study. The rightmost column shows the percent cover based on the Archived (Arch.) annotations. (PDF) [file pone.0130312.s005.pdf]

		Cohen's Kappa (%)										Cover (%)
					Visitors							
	Label	Aut.	ALL. 50%	Host	V1	V2	V3	V4	V5	mean	std	Arch.
**Moorea**	All classes	63.61	75.65	74.78	61.55	64.99	33.2	57.13	52.77	53.93	12.47	N.A.
	Coral	70.79	87.69	91.05	90.34	81.88	78.25	86.24	86.67	84.68	4.68	22.4
	Macro	57.62	72.1	69.34	57.63	65.25	59.4	65.8	54.43	60.5	4.92	6.3
	CCA	58.96	72.35	70.2	45.22	63.13	20.66	43.82	33.13	41.19	15.74	43.0
	Turf	31.36	44.41	43.81	33.9	48.81	18.06	24.51	19.15	28.89	12.78	11.3
	Porites	76.13	90.23	92.53	90.85	75.95	34.23	88.17	90.85	76.01	24.16	11.9
	Pocillopora	74.85	88.89	91.13	90.16	80.11	25.87	85.98	88.89	74.2	27.3	4.4
	Montipora	65.16	89.66	91.1	83.74	28.45	11.07	33.52	67.73	44.9	29.89	2.4
	Pavona	66.26	84.98	90.78	80.63	73.14	-2.01	79.75	85.54	63.41	36.84	1.3
	Acropora	43.32	92.23	92.61	78.75	64.54	60.42	64.26	72.51	68.1	7.4	1.0
**Line-Islands**	All classes	54.56	70.48	73.01	54.22	50.64	55.93	51.99	60.67	54.69	3.91	N.A.
	Coral	68.77	88.33	91.83	88.83	85.62	83.66	85.46	88.95	86.5	2.31	24.9
	Macro	59	71.6	76.79	57.42	61.11	64.11	63.24	57.29	60.63	3.19	16.4
	CCA	53.89	64.33	62.93	51.21	42.18	58.62	48.23	63.15	52.68	8.32	16.3
	Turf	49.2	63	62.51	24.15	24.42	51.83	36.11	49.13	37.13	13.14	31.1
	Acropora	77.8	91.4	95.6	96.06	95.14	94.43	93.53	94.43	94.72	0.94	6.2
	Pocillopora	69.27	91.09	93.68	89.86	87.72	70.69	85.92	85.01	83.84	7.58	5.1
	Porites	47.61	76.22	84.75	78.64	65.67	50.78	44.09	60.75	59.99	13.4	4.0
	Montipora	41.03	84.15	88.15	78.12	67.41	24.16	68.3	70.89	61.78	21.44	3.6
	Favia	22.71	47.52	56.58	54.15	65.06	13.45	71.16	61.6	53.08	22.99	1.8
	Pavona	8.53	59.6	64.74	8.09	9	-1.25	25.23	23.74	12.96	11.27	1.1
	Platygyra	39.82	62.37	77.68	95.21	79.9	78.14	0	89.95	68.64	39.01	0.6
	Millepora	15.31	95.63	100	69.03	0	0	28.2	62.56	31.96	33.04	0.6
**Nanwan Bay**	All classes	44.75	65.01	72.64	50.88	52.41	61.76	58.19	64.9	57.63	5.98	N.A.
	Coral	51.55	81.7	89.37	87.09	76.27	83.23	87.98	90.5	85.01	5.54	28.4
	Macro	13.52	40.54	60.38	26.57	53.23	59.77	38.11	63.76	48.29	15.58	6.5
	CCA	-0.2	14.67	30.3	34.89	20.51	19.84	23.89	29.05	25.63	6.33	3.9
	Turf	47.53	63.55	71.4	30.26	57.53	64.47	47.05	66.92	53.25	14.99	34.7
	Montipora	57.77	82.19	90.34	86.77	49.32	84.39	86.51	87.34	78.86	16.55	16.3
	Pocillopora	34.49	34.24	35.86	31.16	43.29	40.58	84.21	33.54	46.56	21.62	2.8
	Favites	29.99	76.52	83.27	18.48	62.38	37.82	-0.19	41.21	31.94	23.77	2.8
	Favia	13.91	77.78	85.43	69.66	56.48	41.71	37.75	26.58	46.44	16.82	1.9
	Platygyra	26.96	54.53	82.51	81.9	79.67	75.16	76.31	53.56	73.32	11.36	1.7
	Acropora	71.97	71.14	74.76	51.16	37.28	54.4	63.26	45.5	50.32	9.72	0.8
	Porites	-0.09	35.27	37	26.95	10.95	31.81	11.68	35.58	23.4	11.45	0.8
	Millepora	82.9	95.21	95.54	91.02	82.07	92.17	92.7	95.9	90.77	5.19	7.2
**Heron Reef**	All classes	52.9	71.02	73.98	57.64	58.26	67.18	66.36	69.66	63.82	5.5	N.A.
	Coral	62.73	81.35	86.39	77.92	79.89	79.28	78.97	82.35	79.68	1.65	30.8
	Macro	63.86	78.25	77.68	47.59	67.28	74.09	63.84	74.93	65.54	11.06	5.8
	CCA	-0.28	36.1	40.55	27.64	0	24	27.2	34.34	22.64	13.2	1.8
	Turf	47.02	65.25	66.82	42.87	44.92	61.66	58.53	61.63	53.92	9.27	37.1
