# Supplementary material for: Towards Automated Annotation of Benthic Survey Images: Variability of Human Experts and Operational Modes of Automation
Source: PLoS One. 2015 Jul 8;10(7):e0130312. doi: 10.1371/journal.pone.0130312 (PMC4496057; doi:10.1371/journal.pone.0130312)
Supplement: S2 Table — Probabilities that the estimated cover from a set of annotations and for a certain label and location is the same as the cover estimated from the Archived annotations. Probabilities (p-values as estimated from the permutation t-test) in red italics are below 0.05 / 8 = 0.00625, which means that the null hypothesis can be rejected at a 95% confidence level with a Bonferroni correction for eight repeated measurements. This implies that the particular set of annotations is unreliable for cover estimation. For each location, the first four rows are the functional groups: coral, macroalgae, crustose coralline algae (CCA), and turf algae, followed by the dominant coral genera (i.e. with > 10 Archived annotations), and the hydrozoan Millepora if present in that location. The coral genera are ordered by percent cover based on the Archived annotations. Note that coral genera are not included for Heron Reef because the annotations were not resolved to genus level in the original study. Columns ABU and ALL are the Abundance and Alleviate annotation modes respectively, and V1 to V5 are the Visitors. The rightmost column shows the percent cover based on the Archived (Arch.) annotations. (PDF) [file pone.0130312.s006.pdf]

	Label	p-values								Cover (%)
		ABU.	ALL.	Host	V1	V2	V3	V4	V5	Arch.
**Moorea**	Coral	0.7987	0.5065	0.0626	0.6817	0.0958	0.4526	0.4467	0.1456	22.4
	Macro	0.6965	0.0156	*<0.001*	*0.0050*	*<0.001*	0.0089	*<0.001*	0.7334	6.3
	CCA	0.0598	*<0.001*	0.2945	*<0.001*	*<0.001*	*<0.001*	*<0.001*	*<0.001*	43.0
	Turf	0.5231	*<0.001*	*<0.001*	*<0.001*	*0.0044*	*<0.001*	*<0.001*	*<0.001*	11.3
	Porites	0.4010	0.0123	0.0091	0.0870	0.2584	*<0.001*	0.0565	0.1349	11.9
	Pocillopora	0.8580	0.2375	0.5865	0.0382	0.0243	*<0.001*	0.0217	0.3786	4.4
	Montipora	0.8445	0.0468	0.0607	0.0562	0.8676	*<0.001*	0.0847	0.2922	2.4
	Pavona	0.3575	0.5969	0.1874	0.0323	0.3141	*0.0026*	0.3111	0.1240	1.3
	Acropora	0.4003	0.2498	0.2488	0.2171	0.5084	0.2676	0.0396	0.0624	1.0
**Line - Islands**	Coral	0.8440	0.0721	0.9942	*0.0051*	0.0859	*0.0017*	0.0152	0.2423	24.9
	Macro	0.3582	*<0.001*	0.0156	*0.0023*	*<0.001*	*<0.001*	*<0.001*	*0.0022*	16.4
	CCA	0.6061	*<0.001*	*<0.001*	*<0.001*	*<0.001*	0.8527	0.0442	*0.0015*	16.3
	Turf	0.6857	0.5433	*<0.001*	*<0.001*	*<0.001*	0.3095	*<0.001*	*<0.001*	31.1
	Acropora	0.5311	0.6202	0.2237	0.1801	0.0653	0.7445	0.7619	0.3674	6.2
	Pocillopora	0.2625	0.6781	0.4203	0.1687	0.5156	*<0.001*	0.2367	0.0152	5.1
	Porites	0.0495	0.5628	0.9380	0.6827	0.4391	0.0085	0.1424	*<0.001*	4.0
	Montipora	0.3896	0.8291	0.0615	0.3207	0.0486	0.4412	0.8579	0.0858	3.6
	Favia	0.7887	*0.0016*	*0.0051*	0.1556	0.0392	*<0.001*	0.1730	0.5911	1.8
	Pavona	0.6622	0.4894	0.8478	*<0.001*	*<0.001*	0.4094	0.6520	*<0.001*	1.1
	Platygyra	0.2589	*<0.001*	*<0.001*	*<0.001*	0.4973	0.6223	*<0.001*	*<0.001*	0.6
	Millepora	1.0000	*<0.001*	*<0.001*	0.5000	*<0.001*	*<0.001*	0.5794	*0.0007*	0.6
**Nanwan Bay**	Coral	0.1912	0.0159	*<0.001*	*<0.001*	*<0.001*	0.0069	0.0711	*<0.001*	28.4
	Macro	0.2048	*0.0005*	0.0511	*<0.001*	0.4476	0.3926	*<0.001*	0.6726	6.5
	CCA	*<0.001*	*<0.001*	*<0.001*	*<0.001*	*<0.001*	*<0.001*	*<0.001*	*<0.001*	3.9
	Turf	0.4320	*<0.001*	0.0553	*<0.001*	0.2856	*<0.001*	*<0.001*	0.0066	34.7
	Montipora	0.0668	0.2057	0.0185	0.3788	*<0.001*	0.2104	0.2597	0.9316	16.3
	Pocillopora	*<0.001*	*<0.001*	*<0.001*	*<0.001*	*<0.001*	0.8749	0.0378	*<0.001*	2.8
	Favites	0.0289	0.3639	0.2342	*<0.001*	0.0559	0.0461	*<0.001*	0.0121	2.8
	Favia	0.8687	*<0.001*	0.1783	0.0094	0.1186	0.0847	0.0624	0.0468	1.9
	Platygyra	0.2776	0.2962	*0.0021*	*<0.001*	0.6503	*<0.001*	0.0762	0.5183	1.7
	Acropora	0.0370	0.0151	0.0329	*0.0006*	*<0.001*	*0.0040*	0.0773	*<0.001*	0.8
	Porites	1.0000	0.5149	0.9696	0.0728	*0.0021*	0.4472	*<0.001*	*<0.001*	0.8
	Millepora	0.9065	0.0773	0.4270	0.9447	0.2494	0.8868	0.0981	0.7022	7.2
**Heron Reef**	Coral	*0.0039*	0.0298	0.0337	*<0.001*	*<0.001*	*0.0020*	*<0.001*	*<0.001*	30.8
	Macro	0.0668	0.3233	0.3347	*<0.001*	0.1427	0.4842	0.1363	*0.0005*	5.8
	CCA	0.9947	*0.0030*	0.1713	0.0109	*<0.001*	0.8651	*0.0007*	0.0192	1.8
	Turf	*<0.001*	*<0.001*	0.1389	*<0.001*	*0.0016*	*0.0042*	*0.0054*	*<0.001*	37.1
